# Maternal immune activation disrupts the kynurenine pathway of tryptophan metabolism in the placenta–fetal brain axis in rats

**DOI:** 10.1186/s10020-026-01587-1

**Published:** 2026-08-07

**Authors:** Cilia Abad, Morgane Robles, Ramon Portillo, Mia Salma Alsouki, Hana Horackova, Amirehsan Saidi, Veronika Vachalova, Tetiana Synova, Fiona Kumnova, Mohammad Rida Ghaddar, Ivan Vokral, Rona Karahoda, Lukas Lochman, Daniel Heblik, Radim Kucera, Frantisek Staud

**Affiliations:** 1 Department of Pharmacology and Toxicology, Faculty of Pharmacy in Hradec Kralove, Charles University, Hradec Kralove, Czech Republic; 2 UniLaSalle, IDEALISS Research Unit, Rouen, France; 3 Department of Pathology and Laboratory Medicine, The Saban Research Institute, Children Hospital Los Angeles, University of Southern California, Los Angeles, CA 90027 USA; 4 Department of Pharmaceutical Chemistry and Pharmaceutical Analysis, Faculty of Pharmacy in Hradec Kralove, Charles University, Hradec Kralove, Czech Republic

**Keywords:** Maternal immune activation, Kynurenine pathway, Tryptophan metabolism, Placenta, Fetal brain, Neurodevelopment, Sex differences, Quinolinic acid, Kynurenic acid

## Abstract

**Background:**

Maternal immune activation is a major risk factor for neurodevelopmental disorders; however, the molecular mechanisms linking gestational inflammation to fetal brain vulnerability remain incompletely defined. The tryptophan–kynurenine pathway is highly responsive to immune signaling and represents a potential mediator of placenta–fetal brain communication.

**Methods:**

Pregnant rats received Poly I:C (5 or 15 mg/kg) or lipopolysaccharide (1 mg/kg) on gestational days 17–18. Maternal plasma, amniotic fluid, placenta, decidua, and fetal brain were collected 4, 24, and 48 h after the final injection. Cytokines, gene expression, protein levels, and kynurenine-pathway metabolites were quantified, and multivariate analyses were performed to assess temporal and compartment-specific responses.

**Results:**

Maternal inflammation was confirmed by increased IL-6 and IL-1β in plasma, together with elevated CRP and TNF-α in amniotic fluid. The placenta showed rapid remodeling of the kynurenine pathway, including induction of indoleamine 2,3-dioxygenase 1 protein and time-dependent changes in downstream enzymes and in the expression of amino acid transporters. These effects were sex dependent at the protein levels, with female placentas showing increased kynurenine aminotransferase expression, while placental kynurenine and quinolinic acid concentrations increased with treatment independent of sex. The decidua displayed limited and non–sex-dimorphic responses. In the fetal brain, transcriptional changes emerged later and were strongly sex dependent, with broader downregulation of kynurenine-pathway and transporter genes in females. Multivariate analyses indicated that placental responses preceded and were associated with fetal brain transcriptional remodeling.

**Conclusion:**

Following maternal immune activation, the placenta showed early and pronounced remodeling of the kynurenine pathway that preceded and was statistically associated with sex-specific changes in the fetal brain. This temporal and associative pattern identifies placental kynurenine metabolism as a candidate mediator linking maternal inflammation to differential neurodevelopmental vulnerability.

**Supplementary Information:**

The online version contains supplementary material available at https://doi.org/10.1186/s10020-026-01587-1.

## Background

The neurodevelopmental impairment hypothesis (Weinberger 1995) proposes that neurodevelopmental disorders (NDDs), including schizophrenia, autism spectrum disorder (ASD), attention deficit/hyperactivity disorder (ADHD), learning disabilities, and intellectual disability, do not result from late-onset degenerative processes, but rather from early disruptions in brain development, occurring during prenatal or early postnatal life (Rapoport et al. 2012). Among the environmental factors implicated in this disruption, maternal infections during pregnancy have consistently emerged as significant risk factors across both epidemiological and experimental studies (Yates and Mulkey 2024).

A wide range of infections during pregnancy, including viral, bacterial, and protozoan infections, have been associated with an increased risk of NDDs in offspring (Estes and McAllister 2016). The elevated risk does not appear to depend on the specific pathogen, but rather on the maternal immune response, particularly the release of pro-inflammatory cytokines such as IL-6, IL-1β, and TNF-α (Smith et al. 2007). These immunological changes define a state known as maternal immune activation (MIA), which is increasingly recognized as a critical contributor to altered fetal brain development (Estes and McAllister 2016).

Despite the well-established association between MIA and NDDs, the molecular mechanisms linking maternal inflammation to fetal brain disruption remain incompletely understood. One increasingly recognized candidate pathway is the tryptophan–kynurenine metabolic axis, which is particularly sensitive to immune signaling (Cervenka et al. 2024). Pro-inflammatory cytokines, including interferon-gamma (IFN-γ), TNF-α, and IL-6, induce the expression of indoleamine 2,3-dioxygenase (IDO1), the first and rate-limiting enzyme of the kynurenine (KYN) pathway, thereby redirecting tryptophan (TRP) metabolism away from serotonin synthesis toward KYN production (Dehhaghi et al. 2024). KYN is further metabolized along two major branches: one via kynurenine aminotransferases (KATs), producing kynurenic acid (KYNA), a neuroprotective NMDA receptor antagonist; and the other via kynurenine monooxygenase (KMO), leading to 3-hydroxykynurenine and ultimately to quinolinic acid (QUIN), a neurotoxic NMDA receptor agonist (Pocivavsek et al. 2024). These two metabolites, KYNA and QUIN, exert opposing effects on glutamatergic neurotransmission, and their relative balance is critical for normal brain development (Murakami et al. 2023). Immune-mediated dysregulation of the KYN pathway can shift this balance toward increased production of neurotoxic compounds, contributing to excitotoxicity, oxidative stress, and impaired neurodevelopment, all of which have been implicated in the pathophysiology of NDDs (Perez et al. 2012; Savino et al. 2020).

The placenta serves as a dynamic interface between maternal and fetal systems, actively sensing and responding to environmental stressors such as inflammation (Sandovici et al. 2012). Its regulatory functions extend beyond nutrient and oxygen exchange to include immune modulation and metabolic regulation, both essential for healthy fetal development (Woods et al. 2023). Inflammatory disruptions of placental function can alter the local expression of key KYN pathway enzymes, potentially reshaping the production and availability of neuroactive metabolites that influence the developing fetal brain. These insights have led to the emergence of the “placenta–brain axis,” a concept that emphasizes the interdependence between placental metabolic adaptations and fetal neurodevelopment (Rosenfeld 2021).

Recent evidence from our group indicates that maternal inflammation disrupts the placental KYN pathway, potentially altering fetal exposure to neuroactive metabolites. Across clinical, ex vivo, and in vivo models generated within our group, inflammatory conditions consistently shift placental TRP metabolism toward increased KYN pathway activity, accompanied by reduced serotonin synthesis and an imbalance favoring neurotoxic metabolites, particularly an elevated QUIN/KYNA ratio (Karahoda et al. 2021; Cifkova et al. 2024; Abad et al. 2024, 2025). These converging findings support the concept that inflammation-driven reprogramming of placental TRP metabolism may contribute to a neurotoxic intrauterine environment with potential consequences for fetal neurodevelopment.

Despite accumulating evidence that inflammation reprograms placental TRP metabolism, it remains unclear whether these alterations propagate to the fetal brain and reshape local KYN pathway activity. Moreover, the contribution of the decidua, an essential immunoregulatory compartment at the maternal–fetal interface, to this process has not been systematically investigated. In particular, whether decidual responses to inflammation influence placental metabolic adaptation, and whether these effects exhibit sex-dependent and time-dependent dynamics across the placenta–fetal brain axis, remains poorly defined. Addressing these questions requires a systematic evaluation of TRP–kynurenine metabolism across the decidua, placenta, and fetal brain, with particular attention to temporal progression and sex-specific susceptibility.

Due to ethical and technical constraints, mechanistic investigation of MIA during pregnancy relies on preclinical models. Rodents, particularly rats, offer strong translational relevance, recapitulating key neurochemical, transcriptomic, and epigenetic alterations observed in human neurodevelopmental disorders, while their hemochorial placentation enables comparable immune–metabolic interactions at the maternal–fetal interface (Woods et al. 2023; Soares et al. 2018). Among available species, the Wistar rat is especially well suited for studying placental TRP metabolism under inflammatory conditions (Badawy 2015).

To address current gaps in understanding, we employed a late-gestation MIA model triggered by viral- and bacterial-like immune stimuli to determine how systemic inflammation reshapes TRP metabolism along the KYN pathway in the decidua, placenta and the developing fetal brain. By using a time-resolved approach, we aimed to capture the dynamics of sequential molecular responses across the maternal–fetal axis, including transcriptional, protein, and metabolite-level changes. This strategy allows a more accurate representation of how inflammatory signals shape placental and fetal metabolic programming and provides mechanistic insight into how prenatal immune challenges may contribute to altered neurodevelopmental trajectories.

## Methods

### Chemicals and reagents

Lipopolysaccharide (LPS) from *Escherichia coli* O26:B6 was obtained from Sigma-Aldrich (St. Louis, MO, USA). High molecular weight polyinosinic:polycytidylic acid (Poly I:C) was purchased from InvivoGen (San Diego, CA, USA). Reagents for bicinchoninic acid (BCA) protein assay were acquired from Thermo Scientific (Rockford, IL, USA). Tri Reagent® solution for RNA extraction was sourced from Molecular Research Center, Inc. (Cincinnati, OH, USA). Unless otherwise stated, all other reagents and chemicals used in the study were of analytical grade and purchased from standard commercial suppliers.

### Animals

Female Wistar rats were obtained from Velaz (Czech Republic) and housed under standard laboratory conditions (22 ± 2 °C, 50 ± 10% humidity, 12-h light/dark cycle) in the animal facility of the Faculty of Pharmacy, Charles University in Hradec Kralove. After an acclimatization period, females were mated overnight with proven breeder males. The presence of sperm cells in a vaginal swab the following morning was designated as gestational day (GD) 1, marking the beginning of pregnancy. Pregnant rats were housed in groups of 2–3 per cage and had ad libitum access to food and water. All experimental procedures were approved by the Animal Welfare Committee of the Faculty of Pharmacy, Charles University, and authorized by the Czech Ministry of Education, Youth and Sports (MSMT-11891/2023–4), in accordance with Act No. 246/1992 Coll. on the Protection of Animals Against Cruelty.

### Treatment protocol

To induce MIA, pregnant Wistar rats received intraperitoneal (i.p.) injections of either Poly I:C (5 mg/kg or 15 mg/kg) or LPS (1 mg/kg) on GD 17 and 18. Dams were sacrificed at 4, 24, or 48 h following the second injection, corresponding to GD18, GD19, and GD20, respectively. At the time of sacrifice, animals were anesthetized with pentobarbital (40 mg/kg) administered via the tail vein. Following anesthesia, the abdominal cavity was opened, and both uterine horns were carefully excised and examined. The number of live fetuses, resorptions, and dead fetuses was recorded for each dam. Live fetuses were recognizable, viable fetuses; dead fetuses were recognizable but non-viable fetuses showing no signs of viability; and resorptions were implantation sites lacking a recognizable fetus, identified as placental or decidual remnants. Total implantation sites per dam were calculated as the sum of live fetuses, dead fetuses, and resorptions. Post-implantation loss was calculated as [(resorptions + dead fetuses)/total implantation sites] × 100, expressed as a percentage. Litter size refers to the number of live fetuses per dam. Maternal blood and amniotic fluid were collected, and placentas, decidua, and fetal brains were dissected. All collected tissues were snap-frozen and stored at –80 °C for subsequent molecular and biochemical analyses. A total of 12 experimental groups were included (control and three treatment conditions across three time points), with *n = *6–7 dams per group.

### Cytokine analysis

Concentrations of the proinflammatory cytokines interleukin-6 (IL-6), interleukin-1 beta (IL-1β), tumor necrosis factor-alpha (TNF-α), and C-reactive protein (CRP) were quantified in maternal plasma and amniotic fluid using high-sensitivity enzyme-linked immunosorbent assay (ELISA) kits (Thermo Scientific, Rockford, IL, USA). All assays were performed in accordance with the manufacturer’s instructions.

### RNA extraction and cDNA synthesis

Total RNA was extracted from placental, decidual (100 mg), and fetal brain tissues (50 mg) using Tri Reagent Solution (Molecular Research Centre, Cincinnati, OH, USA), according to the manufacturer’s protocol. The quality and purity of RNA were assessed spectrophotometrically using the NanoDrop™ 1000 (Thermo Fisher Scientific, Waltham, MA, USA). The A260/A280 ratio was used to evaluate protein contamination, while the A260/A230 ratio served to assess possible contamination with organic solvents or chaotropic agents. RNA concentrations were determined based on absorbance at 260 nm. First-strand cDNA synthesis was carried out using the iScript™ Advanced cDNA Synthesis Kit (Bio-Rad, Hercules, CA, USA) in a T100™ Thermal Cycler (Bio-Rad), following the manufacturer's instructions.

### Quantitative real-time PCR (qPCR) analysis

Quantitative gene expression analysis was performed using the QuantStudio™ 5 Real-Time PCR System (Thermo Fisher Scientific, Waltham, MA, USA). Reactions were carried out in 5 μL volumes per well, each containing 12.5 ng/μL of cDNA, TaqMan® Universal Master Mix II (without UNG), and predesigned TaqMan® Gene Expression Assays (Thermo Fisher Scientific), following the thermal cycling protocol provided by the manufacturer. All samples were analyzed in technical triplicates. For normalization, a panel of endogenous control genes was used to ensure accurate quantification. In placental and fetal brain tissues, expression levels were normalized against the geometric mean of β2-microglobulin (*B2m*), tyrosine 3-monooxygenase/tryptophan 5-monooxygenase activation protein zeta (*Ywhaz*), TATA-box binding protein (*Tbp*), and glyceraldehyde-3-phosphate dehydrogenase (*Gapdh*). For decidual tissues, normalization was performed using *Gapdh*, *Ywhaz*, and *Tbp* as reference genes. Relative gene expression was calculated using the ΔΔCt method. The TaqMan Gene Expression Assay identifiers for all target and reference genes are listed in Supplementary Table 1.

### Protein extraction and western blot analysis

Placental and decidual tissues were homogenized on ice using a lysis buffer composed of 20 mM Tris–HCl (pH 6.8), 150 mM NaCl, 12.7 mM EDTA, 1 mM EGTA, 4 mM Na₄P₂O₇, 1 mM Na₃VO₄, 1% Triton X-100, and a protease inhibitor cocktail. The homogenates were centrifuged at 10,000 × g for 15 min at 4 °C, and the resulting supernatants were collected and stored at –80 °C until further use. Protein concentrations were determined using the Pierce™ BCA Protein Assay Kit (Thermo Fisher Scientific, Rockford, IL, USA) according to the manufacturer’s instructions.

For western blotting, 80 µg of total protein per sample was mixed with loading buffer, denatured at 70 °C for 10 min, and resolved on 13.5% mPAGE® Lux Bis–Tris polyacrylamide gels using MOPS running buffer. Proteins were transferred onto PVDF membranes (Bio-Rad, Hercules, CA, USA), which were then blocked with TBS-T buffer (20 mM Tris–HCl, 150 mM NaCl, 0.1% Tween-20) containing 5% bovine serum albumin (BSA) for 1 h at room temperature. Membranes were incubated overnight at 4 °C with primary antibodies targeting IDO1 (Thermo Fisher Scientific, PA5-79,437, 1:1000), KMO (Proteintech, 10,698–1-AP, 1:1000), or KAT (Thermo Fisher Scientific, PA5109485, 1:1000). After washing, blots were incubated for 1 h at room temperature with anti-rabbit HRP-conjugated secondary antibody (Dako, P0217, 1:20,000). Immunoreactive bands were detected using ECL™ Prime Western Blotting Detection Reagent and visualized on a ChemiDoc™ MP Imaging System (Bio-Rad). Equal protein loading was verified by probing for β-actin (Abcam, ab8226, 1:10,000) and detection with anti-mouse HRP secondary antibody (Dako, P0260, 1:20,000).

### Quantification of tryptophan and kynurenine pathway metabolites

Placentas were homogenized on ice at 3 mL buffer per g tissue in an ice-cold solution containing 250 mM sucrose, 50 mM Tris–HEPES (pH 7.2), 5 mM EGTA, 5 mM EDTA, and 1 mM PMSF. The homogenate was centrifuged at 800 × g for 10 min at 4 °C. The resulting supernatant was reserved for metabolite quantification. For deproteinization, 150 µL of 25% (w/v) perchloric acid was added to 600 µL of supernatant, mixed, and incubated on ice for 10 min. Precipitated proteins were pelleted by centrifugation at 16,000 × g for 15 min at 4 °C, and the clarified supernatant was collected for HPLC analysis.

Concentrations of TRP, KYN, KYNA, and QUIN were measured in maternal plasma and placental tissue homogenates. TRP, KYN, and KYNA were analyzed using a Shimadzu LC20 high-performance liquid chromatography (HPLC) system (Shimadzu, Japan), equipped with a Kinetex EVO C18 column (150 × 3 mm, 5 µm particle size, 100 Å; Phenomenex, USA) and a guard column. Chromatographic separation was performed at 20 °C with a flow rate of 0.5 mL/min. TRP and KYN were quantified by ultraviolet detection at 289 nm. KYNA was detected fluorometrically using a mobile phase of 97:3 (v/v) zinc acetate (0.05 M with 0.025% acetic acid) and acetonitrile, with excitation/emission wavelengths set at 330/385 nm. QUIN levels were assessed by gas chromatography-mass spectrometry (GC–MS) using deuterated quinolinic acid as an internal standard, following validated analytical procedures.

### Statistical analysis

Statistical analyses were performed using RStudio software (version 2024.04.2 + 764, R software version 4.2.2). Because animals were sacrificed at each time point independently, analyses were conducted separately for each time point (4 h, 24 h, 48 h). Missing values were imputed by Principal Component Analysis (PCA)-based regularisation using the *missMDA* package. Only litters with at least two matched offspring were retained in each tissue-specific dataset.

#### Statistical tools

Permutation-based ANOVAs were performed using the *aovp* function from the *LmPerm* package, and post hoc pairwise permutation tests with false discovery rate (FDR) correction were performed using the *pairwisePermutationTest* function from the *rcompanion* package. PCA and Multiple Factor Analysis (MFA) were performed using the *FactoMineR* package, with variables scaled to unit variance prior to analysis. The corresponding biplots were drawn using the *factoextra* package, with 95% confidence ellipses drawn around each group centroid. To formally test the significance of group separation observed in PCA and MFA ordination plots, permutation-based multivariate analysis of variance (PERMANOVA) was applied using the *PERMANOVA* package. Boxplots were generated using the *ggplot2* package. Z-score-normalised heatmaps were generated using the *ComplexHeatmap* package; within each group or sex, samples were ordered from low to high mean z-score, row clustering was performed using the default hierarchical method, and column order was fixed by the within-group sorting. A significance threshold of p ≤ 0.05 was applied throughout.

#### Biometrics and placental phenotype

The effects of treatment group, fetal sex, and litter size on fetal weight, placental weight, placental efficiency, and brain-to-body weight ratio were assessed by permutation-based ANOVA. No significant Group × Sex interaction was detected; therefore, only main effects were retained in the final models. The litter was included as an error stratum to account for the non-independence of littermates. Pairwise post-hoc tests with FDR correction were applied when a main effect reached significance. Significant results are displayed as boxplots.

#### Inflammatory cytokines

*Maternal plasma.* Cytokine concentrations were compared across treatment groups using single-factor permutation-based ANOVA, followed by pairwise post-hoc tests with FDR correction. Results are presented as boxplots.

*Amniotic fluid, decidua, placenta and fetal brain.* Cytokine concentrations in amniotic fluid and cytokine gene expression in decidua, placenta, and fetal brain were analysed using permutation-based ANOVA including treatment group, fetal sex, and their interaction as fixed factors, with litter as an error stratum. Litter size and fetal weight were evaluated as potential covariates but were not significant and therefore not retained in the final models. Pairwise post-hoc tests with FDR correction were applied on significant effects. Significant results are presented as boxplots or heatmaps.

*Multiple factor analysis*. To obtain a broader view of the inflammatory response and its coordination across compartments, an MFA was performed integrating five variable blocks: maternal plasma cytokine concentrations, amniotic fluid cytokine concentrations, and gene expression data from decidua, placenta, and fetal brain. Group separation was formally tested by PERMANOVA.

#### Gene expression in fetal brain, placenta and decidua

For each tissue and time point, gene expression was analyzed using two complementary approaches. First, PCA was applied to the kynurenine-pathway genes (*Ido2*, *Kmo*, *Kynu*, *Haao*, *Qprt*, *Kat1*) and System L transporter genes (*Slc3a2*, *Slc7a5*, *Slc7a8*) to visualize overall differences between treatment groups, and group separation was formally tested by PERMANOVA. Second, each gene was modeled individually using permutation-based ANOVA. Treatment group and fetal sex were included as fixed effects; the Group × Sex interaction term was retained when significant. The litter was included as an error stratum. Litter size, fetal weight, placental weight, and fetal brain weight were evaluated as covariates and retained only when significant. P-values were FDR-corrected to adjust for multiple comparisons across genes, and pairwise post-hoc tests with FDR correction were applied on significant effects. Significant results are presented as boxplots, heatmaps, or PCA biplots colored by sex.

#### Within-litter placenta–fetal brain correlation

To assess whether placental and fetal-brain kynurenine-pathway transcripts co-vary within litters independently of the maternal immune stimulus, the six genes quantified in both compartments (*Ido2*, *Kmo*, *Kynu*, *Haao*, *Qprt*, and *Kat1*) were analyzed as matched placenta–brain pairs. Placental and fetal-brain samples sharing the same litter and fetal-sex identifier were paired, yielding 150 pairs from 78 litters across the three post-injection time points. For each gene, the association between placental and fetal-brain transcript levels was quantified as a partial Spearman rank correlation adjusting for treatment group, time point (4, 24, and 48 h), and fetal sex. Statistical significance was assessed non-parametrically using a restricted permutation procedure (10,000 permutations) in which whole litters were exchanged as blocks, keeping the two fetuses of a litter together. Permutation p-values were adjusted across the six genes using the Benjamini–Hochberg false discovery rate (FDR), with q < 0.05 considered significant. As a supporting analysis, partial correlations were additionally estimated within each treatment group, adjusting for time and sex.

#### Placental protein expression

Placental protein concentrations (IDO1, KMO, KAT) were analyzed by permutation-based ANOVA with litter as an error stratum. Analyses were subsequently conducted separately in males and females to characterize sex-specific responses. Pairwise post-hoc tests with FDR correction were applied on significant effects, and results are presented as sex-stratified heatmaps.

#### Plasma and placental metabolites

Placental concentrations of TRP pathway metabolites (TRP, KYN, KYNA, QUIN) were analyzed by permutation-based ANOVA, including treatment group, fetal sex, and their interaction as fixed factors, with FDR correction. Significant results are presented as boxplots. To explore coordinated variation across the kynurenine pathway, an MFA was performed integrating placental gene expression (*Ido2*, *Kat1*, *Kmo*), placental protein expression (IDO1, KAT, KMO), placental metabolite concentrations, and maternal plasma metabolite concentrations.

## Results

### Maternal immune activation induces systemic and compartment-specific inflammatory responses during late gestation

**Fig. 1:**
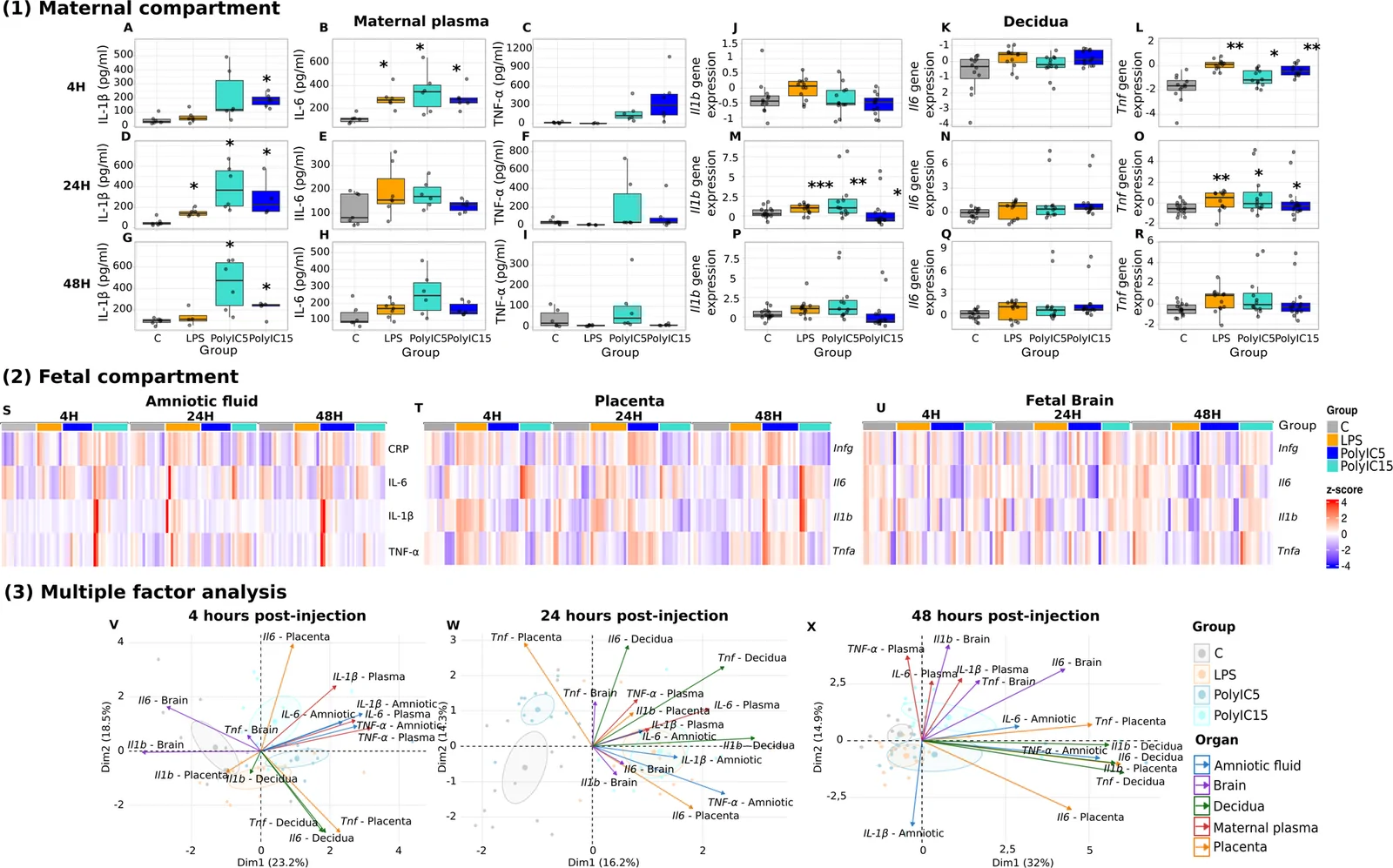
Maternal immune activation induces temporally structured and compartment-specific inflammatory responses across the maternal–fetal axis. MIA was induced by intraperitoneal administration of Poly I:C (5 or 15 mg/kg) or LPS (1 mg/kg) on gestational days 17–18, and inflammatory responses were assessed at 4, 24, and 48 h after the final injection. (A–I) Maternal plasma concentrations of pro-inflammatory cytokines IL-1β, IL-6, and TNF-α measured at each time point, confirming systemic immune activation. (J–R) Decidual gene expression of pro-inflammatory cytokines (*Tnf*, *Il1b*, and *Il6*), showing a transient and stimulus-dependent inflammatory response at the maternal–fetal interface. (S) Heatmap of pro-inflammatory cytokine protein concentrations (CRP, IL-6, IL-1β, TNF-α) in amniotic fluid, illustrating dynamic inflammatory exposure within the fetal compartment. (T) Heatmap of placental cytokine gene expression (*Ifng*, *Il6*, *Il1b*, and *Tnf*), demonstrating temporally structured and stimulus-specific transcriptional activation in response to maternal immune challenge. (U) Heatmap of fetal brain cytokine gene expression (*Ifng*, *Il6*, *Il1b*, and *Tnf*), revealing delayed neuroinflammatory responses relative to maternal and placental compartments. (V–X) Multiple factor analysis integrating cytokine data across maternal plasma, decidua, amniotic fluid, placenta, and fetal brain at 4 h (V), 24 h (W), and 48 h (X). MFA highlights the temporal progression from early maternal and extra-fetal inflammatory signaling to coordinated activation at the maternal–fetal interface and selective engagement of fetal brain inflammatory markers at later time points. Ellipses represent group-level confidence regions. Statistical analyses are described in the Methods. *p < 0.05, ** *p* < 0.01 and, ***p < 0.001 vs control

MIA was induced by intraperitoneal administration of Poly I:C (5 or 15 mg/kg) or LPS (1 mg/kg) on GD17–GD18 and validated by quantifying maternal plasma IL-1β, IL-6, and TNF-α. To map how this inflammatory response was distributed across the maternal–fetal unit, we next profiled cytokine signals at the maternal interface (decidua), within the fetal environment (amniotic fluid), and in fetal tissues (placenta and fetal brain) using protein and transcript level readouts (Fig. 1).

In maternal plasma, MIA elicited a rapid and stimulus-dependent cytokine response. IL-6 levels increased early following all immune challenges, indicating a robust systemic inflammatory activation. TNF-α showed a significant overall group effect at 4 h without significant pairwise differences relative to controls. In contrast, IL-1β induction was more selective, with an early increase observed after Poly I:C 15 mg/kg. At later time points, IL-1β elevations persisted across treatments, remaining increased at 24 h following both LPS and Poly I:C (5 and 15 mg/kg) exposure, and extending to 48 h after Poly I:C administration (Fig. 1A-I).

At the maternal–fetal interface, decidual cytokine gene expression exhibited a temporally restricted inflammatory response following MIA. At 4 h, *Tnf* expression was significantly increased across all treatment groups, whereas *Il1b* and *Il6* showed overall group effects without significant pairwise differences relative to controls. By 24 h, the inflammatory response became more pronounced, with *Il1b* and *Tnf* upregulated across all treatment groups, while *Il6* remained unchanged. At 48 h, no significant differences were detected for any cytokine transcripts (Fig. 1J–R). A sex × treatment interaction was detected for *Il1b* at 4 h, driven by increased expression in female-derived deciduas following LPS exposure (Supplementary Fig. 1).

Analysis of amniotic fluid cytokine concentrations revealed a dynamic inflammatory response within the fetal compartment following MIA. At 4 h, CRP and TNF-α concentrations increased following Poly I:C 5 mg/kg. By 24 h, TNF-α concentrations were increased in the LPS and Poly I:C 5 mg/kg groups. At 48 h, CRP levels were elevated following Poly I:C administration at both doses, indicating sustained inflammatory signaling within the amniotic environment (Fig. 1S; Supplementary Table 2). A sex × treatment interaction was formally tested for each amniotic fluid cytokine, with significant interactions detected for CRP and TNF-α at 4 h and for CRP and IL-6 at 48 h. At 4 h, CRP concentrations increased in female-derived samples following LPS and Poly I:C 5 mg/kg exposure. TNF-α levels were elevated in females treated with LPS and Poly I:C 15 mg/kg, whereas in male-derived samples TNF-α increased following Poly I:C 5 mg/kg. No sex-dependent differences were detected at 24 h. At 48 h, CRP concentrations were increased in female-derived samples following LPS and Poly I:C 15 mg/kg treatment. For IL-6 at 48 h, the significant interaction was not accompanied by significant pairwise comparisons (Supplementary Fig. 2).

Placental cytokine gene expression displayed a temporally and stimulus-dependent inflammatory profile following MIA. At 4 h, *Il1b* and *Tnf* expression were significantly increased following LPS and Poly I:C 15 mg/kg exposure, while *Ifng* expression was significantly increased following LPS exposure. No significant changes were detected for *Il6* at this time point. By 24 h, *Il1b* expression remained elevated in the LPS group, whereas *Il6* and *Ifng* showed a significant overall group effect that did not reach statistical significance in post hoc comparisons. No significant changes were observed for *Tnf.* At 48 h, no significant differences were detected for any cytokine transcripts (Fig. 1T; Supplementary Table 3). Formal testing revealed no significant sex × treatment interaction and no main effect of sex for any placental cytokine transcript at any time point; accordingly, combined-sex data are presented, with the corresponding sex-stratified expression provided in Supplementary Fig. 3.

In the fetal brain, inflammatory gene expression responses were absent at early time points and emerged only at later stages following MIA. No significant changes were detected at 4 or 24 h for any cytokine transcripts. By 48 h, *Tnf* and *Ifng* expression were significantly decreased following LPS exposure, whereas no changes were observed under Poly I:C treatment. No significant alterations were detected for *Il1b* or *Il6* at any time point (Fig. 1U; Supplementary Table 3). Sex-stratified analysis further revealed a selective and time-dependent sensitivity of the fetal brain to MIA (Supplementary Fig. 4). At 24 h, a significant sex × treatment interaction was detected for *Il6*, driven by reduced expression in female-derived samples following Poly I:C 15 mg/kg exposure, whereas no corresponding changes were observed in males. By 48 h, sex-dependent effects became more pronounced. A significant interaction was observed for *Il1b* and *Ifng*, with reduced expression detected in females following LPS exposure, while no changes were observed in males. *Il6* also showed a trend toward a sex-dependent response at 48 h, with decreased expression in female-derived samples following LPS treatment and no corresponding effect in males. *Tnf* expression was reduced in both sexes following LPS exposure; however, the magnitude and consistency of this response were greater in females, indicating a stronger female-biased sensitivity.

To integrate cytokine responses across maternal, placental, and fetal compartments, MFA was performed at 4, 24, and 48 h, with group separation formally tested using PERMANOVA. At 4 h, the first two MFA dimensions explained 41.6% of total inertia (Dim.1 = 23.2%, Dim.2 = 18.5%), with separation driven primarily by maternal plasma and amniotic fluid cytokines and limited contribution from fetal brain variables (Fig. 1V). PERMANOVA confirmed a significant effect of treatment group (F = 4.53, R^2^ = 0.308, *p* = 0.001), with all three treatment groups significantly displaced from controls and displacement increasing as a function of Poly I:C dose (LPS: Ed = 1.73, *p* = 0.002; Poly I:C 5 mg/kg: Ed = 2.05, *p* = 0.002; Poly I:C 15 mg/kg: Ed = 2.64, *p* = 0.002).

By 24 h, the multivariate structure was more diffuse (Dim.1 = 16.2%, Dim.1 + 2 = 30.5%), reflecting a more distributed inflammatory response across compartments. Cytokine profiles across maternal plasma, decidua, amniotic fluid, and placenta nevertheless became more tightly coordinated, with group discrimination reflecting both overall inflammatory intensity and stimulus-specific transcriptional signatures at the maternal–fetal interface (Fig. 1W). PERMANOVA confirmed significant effects of group (F = 3.63, R^2^ = 0.268, *p* = 0.001), with all treatment groups remaining significantly separated from controls, greater overall displacement than at 4 h, and an attenuated gradient between groups (LPS: Ed = 2.64, *p* = 0.003; Poly I:C 5 mg/kg: Ed = 2.48, *p* = 0.003; Poly I:C 15 mg/kg: Ed = 2.05, *p* = 0.005).

At 48 h, the two MFA dimensions accounted for the highest proportion of total inertia of the three time points (Dim.1 = 32.0%, Dim.1 + 2 = 46.9%), with dominant contributions from placental, decidual, and amniotic cytokines and selective, treatment-dependent engagement of fetal brain inflammatory markers (Fig. 1X). PERMANOVA confirmed a significant overall group effect (F = 2.90, R^2^ = 0.268, *p* = 0.005); however, treatment groups showed substantial overlap with each other and with controls, and displacement from controls was modest for LPS and Poly I:C 5 mg/kg (LPS: Ed = 1.27, *p* = 0.039; Poly I:C 5 mg/kg: Ed** = **1.28, *p* = 0.006), consistent with partial resolution of the inflammatory signature by this time point. Poly I:C 15 mg/kg showed a numerically greater displacement (Ed = 2.37) that did not reach significance after FDR correction (*p* = 0.055), likely reflecting reduced statistical power from the limited number of complete observations at this time point.

Collectively, these results demonstrate a temporally structured and compartment-specific inflammatory response to MIA, characterized by early systemic and placental activation, transient responses at the maternal–fetal interface, and delayed, sex-dependent inflammatory signaling in the fetal brain (Fig. 1A–X).

### Maternal immune activation alters fetal growth trajectories and placental efficiency in a time-dependent manner

**Fig. 2:**
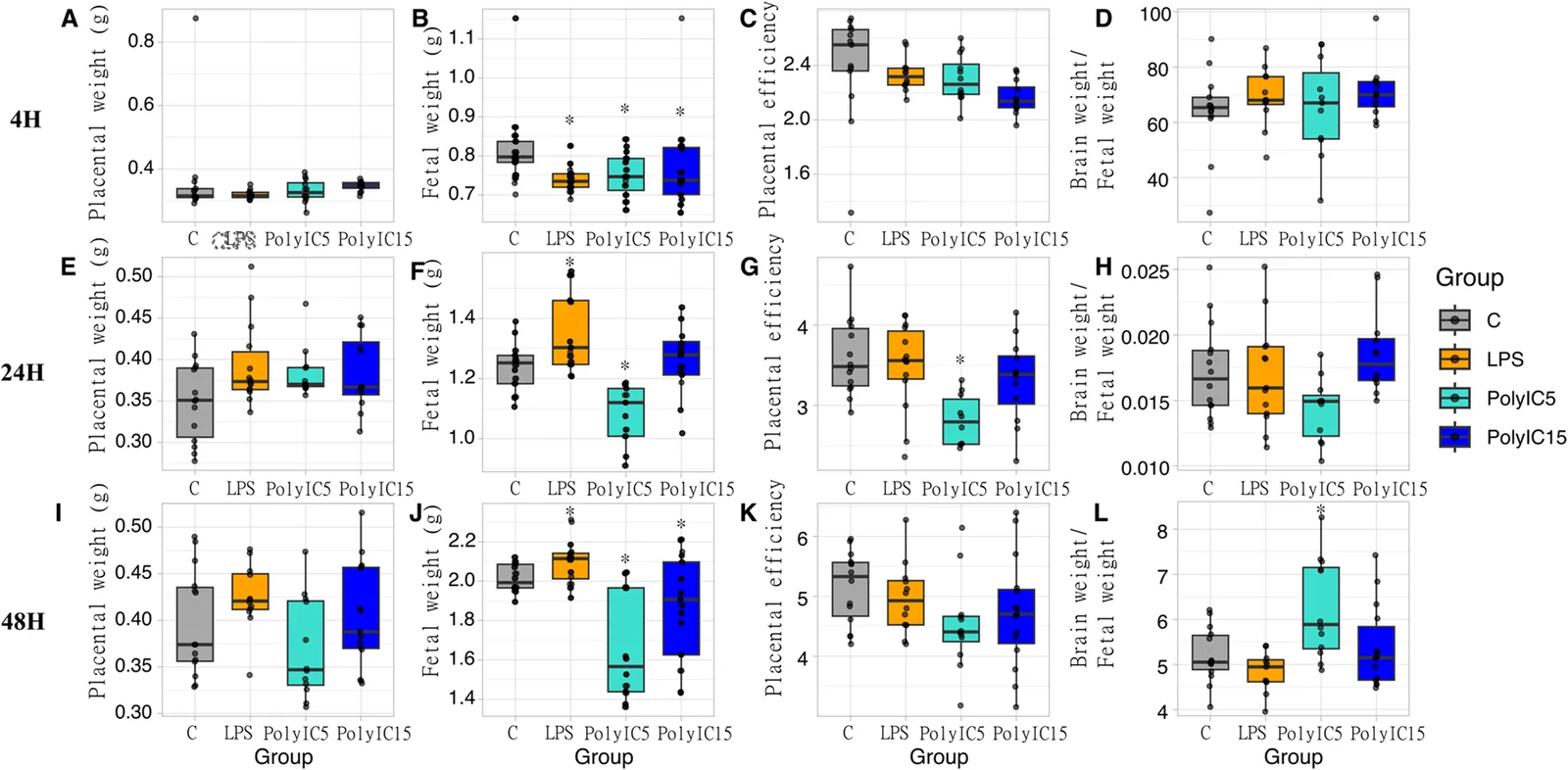
Maternal immune activation induces time-dependent alterations in fetal growth and placental efficiency. Placental weight (**A**, **E**, **I**), fetal weight (**B**, **F**, **J**), placental efficiency (fetal weight/placental weight; **C, G, K**), and brain-to-fetal weight ratio (**D**, **H**, **L**) were assessed at 4, 24, and 48 h following MIA induced by Poly I:C (5 or 15 mg/kg) or LPS (1 mg/kg). Data are shown for control, LPS, Poly I:C 5 mg/kg, and Poly I:C 15 mg/kg groups. Box plots display the median with interquartile range (25th–75th percentile). Statistical comparisons were performed as described in the Methods. *p < 0.05, ** *p* < 0.01, *** *p* < 0.001, ****p < 0.0001 vs control

MIA induced time-dependent alterations in fetal growth and placental efficiency, while placental weight remained largely unchanged across treatments (Fig. 2). At 4 h, fetal weight was significantly reduced in all MIA groups relative to controls, whereas placental weight, placental efficiency, and the brain-to-fetal weight ratio were not significantly affected. By 24 h, divergent growth trajectories emerged; fetal weight was increased following LPS exposure but reduced in the Poly I:C 5 mg/kg group, accompanied by a corresponding decrease in placental efficiency. At 48 h, fetal weight remained elevated in the LPS group but was reduced in both Poly I:C groups. Notably, the brain-to-fetal weight ratio was increased at 48 h following Poly I:C 5 mg/kg exposure (Fig. 2A–L).

Litter composition and pregnancy outcome were assessed across treatment groups (Supplementary Table 4). Live litter size did not differ significantly between groups at any timepoint. Post-implantation loss was low in control and LPS-exposed dams, whereas it was significantly elevated in the Poly I:C 5 mg/kg group at 48 h (Supplementary Fig. 5). Litter size was included as a covariate in all biometric models and was frequently a significant predictor, including for fetal weight, whereas fetal sex and the group × sex interaction were not significant for any parameter; the treatment effect on fetal weight remained significant after adjustment for litter size.

Taken together, these findings indicate that MIA produces a stimulus-divergent trajectory of fetal growth, an early shared reduction in fetal weight followed by opposing LPS- and Poly I:C-associated patterns, with evidence of asymmetric growth restriction emerging under Poly I:C 5 mg/kg by 48 h.

### Maternal immune activation reprograms systemic tryptophan–kynurenine metabolism

Maternal plasma TRP concentrations were not altered at 4 or 24 h but decreased at 48 h following LPS administration (Table 1). At 4 h, KYN levels showed a significant group effect, with increased concentrations following Poly I:C 15 mg/kg, whereas a trend was observed for Poly I:C 5 mg/kg. KYNA concentrations were not significantly altered at this time point. At 24 h, KYN levels remained elevated following Poly I:C 5 mg/kg, while KYNA showed a significant group effect but did not reach statistical significance in post hoc comparisons, with a trend observed for Poly I:C 15 mg/kg. At 48 h, KYN concentrations were increased following Poly I:C 5 mg/kg, while KYNA levels were elevated following LPS exposure. QUIN concentrations remained unchanged across all time points.

**Table 1:** Maternal plasma concentrations of tryptophan and kynurenine-pathway metabolites following maternal immune activation

Metabolite	4 h				24 h				48 h			
	**CTRL**	**Poly I:C 5 mg/kg**	**Poly I:C 15 mg/kg**	**LPS 1 mg/kg**	**CTRL**	**Poly I:C 5 mg/kg**	**Poly I:C 15 mg/kg**	**LPS 1 mg/kg**	**CTRL**	**Poly I:C 5 mg/kg**	**Poly I:C 15 mg/kg**	**LPS 1 mg/kg**
TRP [ng/ml]	8751(7596–8939)	7658 (7264–8906)	9555 (9110–10008)	7250 (6692–8081)	6403 (5423–7631)	6200 (5246–8615)	5911 (4787–6835)	6236(6055–6309)	7691(5059–8205)	6457 (5100–7080)	4833 (4378–7431)	4841 (3212–4980)^**^
KYN [ng/ml]	331 (290–396)	498.5 (408.3–850)	657 (513.8–781.5)^*^	383 (310.3–425.8)	381 (288–453)	527.5 (385.5–1155)^*^	391 (331.8–494)	279 (202–306)	346 (265–392)	540.5 (399.5–645)^**^	400 (335–418)	264 (211–288)
KYNA [ng/ml]	26.4 (22.7–29.4)	28.5 (23.9–44.73)	46.8 (37.63–52.88)	34.3 (24.83–66.2)	22.5 (19.5–29.5)	45.05 (31.98–82.23)	36.4 (22.85–72.9)	24.6 (17.8–28)	21.4 (17.4–33)	36.2 (26.48–41.15)	30.8 (25–42.2)	31.9 (19.6–44.8)^**^
QUIN [ng/ml]	119.4 (102.4–159.3)	93.99 (85.41–205.2)	180 (126.7–236.2)	62.6 (50.35–148)	179.1 (101.4–280.2)	154.9 (107.5–295.4)	81.62 (66.46–138.5)	97.05 (86.1–138.1)	147.5 (97.95–156)	138.4 (130.7–154.4)	150.5 (110.6–276.9)	176.5 (108–210.3)
KYN/TRP Ratio × 10^3^	41.72 (38.04–49.2)	59.18 (52.93–116.1)	67.67 (51.1–86.32)	52.44 (42.92–65.54)	46.92 (38.79–68.19)	88.76 (69.89–122.1)^*^	71.19 (65.92 −96.03)^*^	47.82 (33.19–49.82)	50.32 (38.22–68.06)	95.78 (75.7–103.4)^**^	81.61 (81.61–91.53)^*^	68.94 (43.59–92.51)^*^
KYNA/KYN Ratio × 10^3^	71.59 (64.07–105.1)	66.71 (62.52–84.18)	73.19 (55.82–97.7)	91.64 (66.88–164.6)	69.65 (59.13–114.2)	75.19 (64.89–102.7	100.6 (51.09–157.7)	88.44 (59.82–102.1)	72.14 (65.79–91.42)	60.62 (46.98–96.38)	80.39 (62.11–87.17)	125 (92.76–155.5)^**^
Quin/KYN Ratio × 10^3^	356.4 (309.3–556.3)	212.8 (153.5 −242)	247.7 (217.3–311.2)	175.1 (131.9–396.6)	618 (342.6–933.5)	301.8 (206.3–416.7)^*^	190.6 (176.2–261)^**^	430.5 (317.2–492.2)	410.5 (283.1–582.3)	265 (213.3–391.8)	376.2 (313.4–587.2)	466.3 (375.2–836.5)
QUIN/KYNA Ratio × 10^3^	5007 (4006–5844)	3195 (2488–3511)^*^	4044 (2679–5101)	2098 (1392–2604)^**^	7383 (4648–1359)	3766 (2398–5078)^*^	2425 (1632–3805)^**^	4992 (3570–5595)	5747 (4408–8462)	4384 (3371–5087)	4680 (3596–8949)	4087 (2412–9018)

Maternal plasma concentrations of tryptophan (TRP), kynurenine (KYN), kynurenic acid (KYNA), and quinolinic acid (QUIN), as well as derived metabolite ratios (KYN/TRP, KYNA/KYN, QUIN/KYN, and QUIN/KYNA), were assessed at 4, 24, and 48 h after the final maternal injection of Poly I:C (5 or 15 mg/kg) or LPS (1 mg/kg). Data are presented as median with interquartile range (25th–75th percentile). Statistical comparisons were performed as described in the Methods. Significant differences relative to the control group are indicated by *p < 0.05, **p < 0.01

To assess relative enzymatic activity within the pathway, metabolite ratios were calculated. At 4 h, no significant changes were detected for KYN/TRP, KYNA/KYN, or QUIN/KYN ratios; however, the QUIN/KYNA ratio was significantly reduced following LPS and Poly I:C 5 mg/kg. At 24 h, the KYN/TRP ratio showed a significant group effect, although post hoc comparisons indicated only trends for both Poly I:C doses. In contrast, the QUIN/KYN ratio and the QUIN/KYNA ratio were both reduced following both Poly I:C doses. At 48 h, the KYN/TRP ratio was increased across treatments, reaching significance for all treatment groups. The KYNA/KYN ratio was also increased following LPS, whereas no significant changes were observed for QUIN/KYN or QUIN/KYNA ratios.

Overall, these results indicate a dynamic and time-dependent modulation of systemic TRP metabolism following MIA, characterized by early activation of the KYN pathway and subsequent redistribution of downstream metabolite flux. All maternal plasma metabolite data are summarized in Table 1.

### Principal component analysis reveals tissue-specific and time-dependent reorganization of the kynurenine pathway following maternal immune activation

**Fig. 3:**
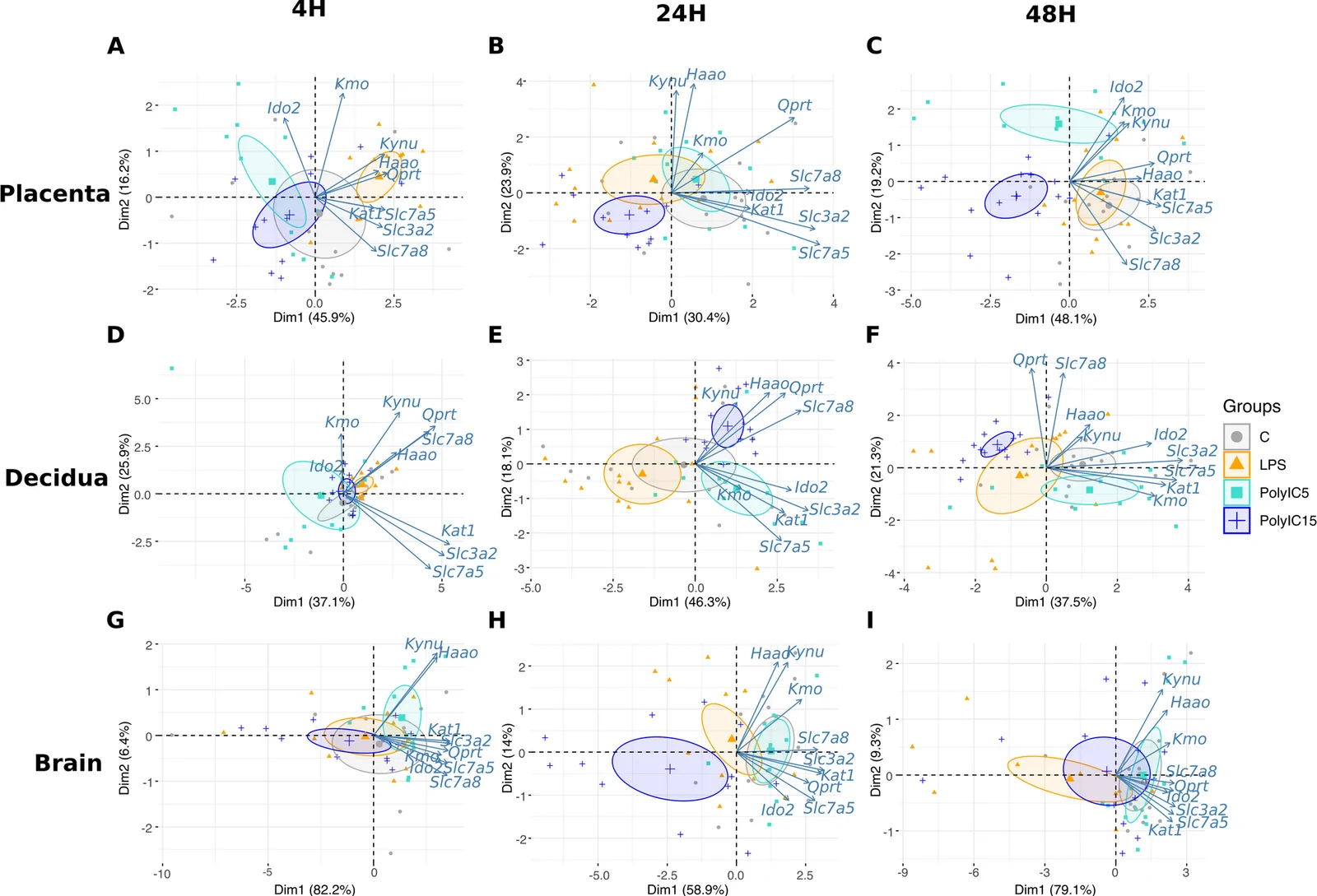
Kynurenine-pathway and System L transporter gene expression in placenta, decidua, and fetal brain following MIA. PCA was performed on normalized gene expression of kynurenine-pathway genes (*Ido2*, *Kmo*, *Kynu*, *Haao*, *Qprt*, *Kat1*) and large neutral amino acid transporters (*Slc3a2*, *Slc7a5*, *Slc7a8*). Panels are arranged by tissue; placenta (**A**–**C**), decidua (**D**–**F**), fetal brain (**G**–**I**) and by time after MIA: 4 h (**A**, **D**, **G**), 24 h (**B**, **E**, **H**), 48 h (**C**, **F**, **I**). Points represent sex-specific pooled samples per litter. Ellipses indicate 95% confidence intervals for each treatment group. Arrows depict variable loadings, indicating the direction and relative contribution of each gene or transporter to the principal components. The percentage of variance explained by PC1 and PC2 is shown on each axis. Treatments included Control, Poly I:C 5 mg/kg, Poly I:C 15 mg/kg, and LPS 1 mg/kg, as indicated by the in-panel color and symbol key

Following MIA, coordinated changes in kynurenine-pathway enzymes and System L transporter genes (Slc7a5/LAT1, Slc7a8/LAT2, and Slc3a2) were evident across tissues, with multivariate expression patterns that differed by treatment and time. PCA of kynurenine-pathway genes (*Ido2*, *Kmo*, *Kynu*, *Haao*, *Qprt*, *Kat1*) and System L transporter genes (*Slc3a2*, *Slc7a5*, *Slc7a8*) revealed distinct, tissue-specific clustering in the placenta, decidua, and fetal brain (Fig. 3).

The most pronounced group-level separation was observed in the placenta, where clustering progressively diverged from controls over time and was strongest at 48 h. In this tissue, treatment groups separated primarily along PC1 (accounting for ~ 30–48% of variance across time points). Displacement from controls shifted over time: LPS showed the greatest separation at 4 and 24 h, whereas both Poly I:C doses showed the greatest displacement by 48 h. Loadings indicated that this separation was driven predominantly by coordinated variation in core kynurenine-pathway enzymes, with prominent contributions from KMO-branch components (*Kmo*, *Kynu*, *Haao*) together with additional contributions from System L transporter genes (*Slc3a2*, *Slc7a5*, *Slc7a8*), consistent with a restructuring of TRP metabolic routing. PERMANOVA confirmed a significant effect of treatment group at 4 h (F = 4.55, R^2^ = 0.235, *p* = 0.001), 24 h (F = 3.85, R^2^ = 0.196, *p* = 0.001) and 48 h (F = 6.79, R^2^ = 0.294, *p* = 0.001). At 4 h, LPS and Poly I:C 5 mg/kg were both significantly separated from controls, though with markedly different displacement magnitudes (LPS: Ed = 2.02, *p* = 0.004; Poly I:C 5 mg/kg: Ed = 0.97, *p* = 0.030). At 24 h, LPS maintained a comparable displacement from controls (Ed = 1.91, *p* = 0.006), whereas Poly I:C 15 mg/kg emerged as significantly separated (Ed = 0.71, *p* = 0.003). At 48 h, the displacement pattern shifted, with both Poly I:C (5 mg/kg and 15 mg/kg) showing the greatest separation from controls (Ed = 2.74, *p* = 0.002 and Ed = 2.94, *p* = 0.002, respectively), whereas LPS displacement was markedly reduced (Ed = 0.44, *p* = 0.039).

In the decidua, PCA revealed more modest separation at 4 h, with clearer divergence emerging at 24 and 48 h, driven primarily by LPS and Poly I:C 15 mg/kg exposure. The relative proximity of decidual clusters to the origin suggests a more limited metabolic response compared with the placenta. PERMANOVA confirmed a significant effect of treatment group at 4 h (F = 2.03, R^2^ = 0.121, *p* = 0.010), 24 h (F = 5.02, R^2^ = 0.236, *p* = 0.001) and 48 h (F = 4.70, R^2^ = 0.225, *p* = 0.001). At 4 h, despite a significant global group effect, no pairwise separation from controls survived FDR correction. At 24 h, Poly I:C 15 mg/kg was the first group to separate significantly from controls (Ed = 1.72, *p* = 0.015), followed by LPS (Ed = 1.81, *p* = 0.015). At 48 h, both LPS and Poly I:C 15 mg/kg maintained significant separation from controls (LPS: Ed = 1.81, *p* = 0.029; Poly I:C 15 mg/kg: Ed = 2.50, *p* = 0.003), with Poly I:C 15 mg/kg showing the greater displacement.

In the fetal brain, clustering remained close to controls at 4 h but diverged markedly at 24 and 48 h, with Poly I:C 15 mg/kg driving the largest separation at 24 h and LPS emerging as the dominant driver by 48 h. Separation along PC1, which explained a large proportion of variance at later time points, was associated with combined variation in kynurenine-pathway enzymes and System L transporter genes. PERMANOVA confirmed these observations, showing no significant group differences at 4 h, but significant effects emerging at 24 h (F = 7.04, R^2^ = 0.266, *p* = 0.001) and 48 h (F = 4.48, R^2^ = 0.197, *p* = 0.002). At 24 h, LPS and Poly I:C 15 mg/kg were both significantly separated from controls, with distinct displacement magnitudes (LPS: Ed = 1.39, *p* = 0.024; Poly I:C 15 mg/kg: Ed = 3.60, *p* = 0.003). At 48 h, only LPS remained significantly separated from controls (Ed = 2.94, *p* = 0.027), whereas Poly I:C 15 mg/kg displacement was no longer significant after FDR correction.

Across tissues, control samples clustered near the origin and displayed limited dispersion, whereas immune-challenged groups showed broader confidence ellipses, reflecting increased coordinated variability in pathway-level expression. Together, these multivariate analyses complement the univariate results and demonstrate that MIA induces structured, tissue- and time-dependent reorganization of the KYN pathway, with the placenta emerging as the primary site of early metabolic divergence and the fetal brain exhibiting delayed secondary metabolic remodeling.

### Maternal immune activation differentially modulates kynurenine-pathway gene, protein, and metabolite profiles across the decidua and placenta

Building on the multivariate patterns identified by PCA, we next performed targeted analyses to quantify treatment-induced changes in individual kynurenine-pathway genes, System L transporter genes, and downstream metabolites relative to controls.

In the decidua, gene expression analysis revealed a restricted and delayed transcriptional response to MIA (Supplementary Fig. 6; Supplementary Table 5). No significant changes were detected at 4 h for any kynurenine-pathway enzymes or System L transporter genes across any treatment group. At 24 h, Poly I:C 15 mg/kg selectively increased the expression of *Qprt* and *Slc7a8*, whereas at 48 h, both Poly I:C 15 mg/kg and LPS were associated with downregulation of *Slc7a5*, alongside reduced *Kmo* expression following Poly I:C 15 mg/kg. PCA and PERMANOVA analyses showed no evidence of sex-dependent clustering or global sex effects. Consistent with these findings, protein expression of IDO1, KAT, and KMO remained unchanged across all conditions (Supplementary Fig. 6).

**Fig. 4:**
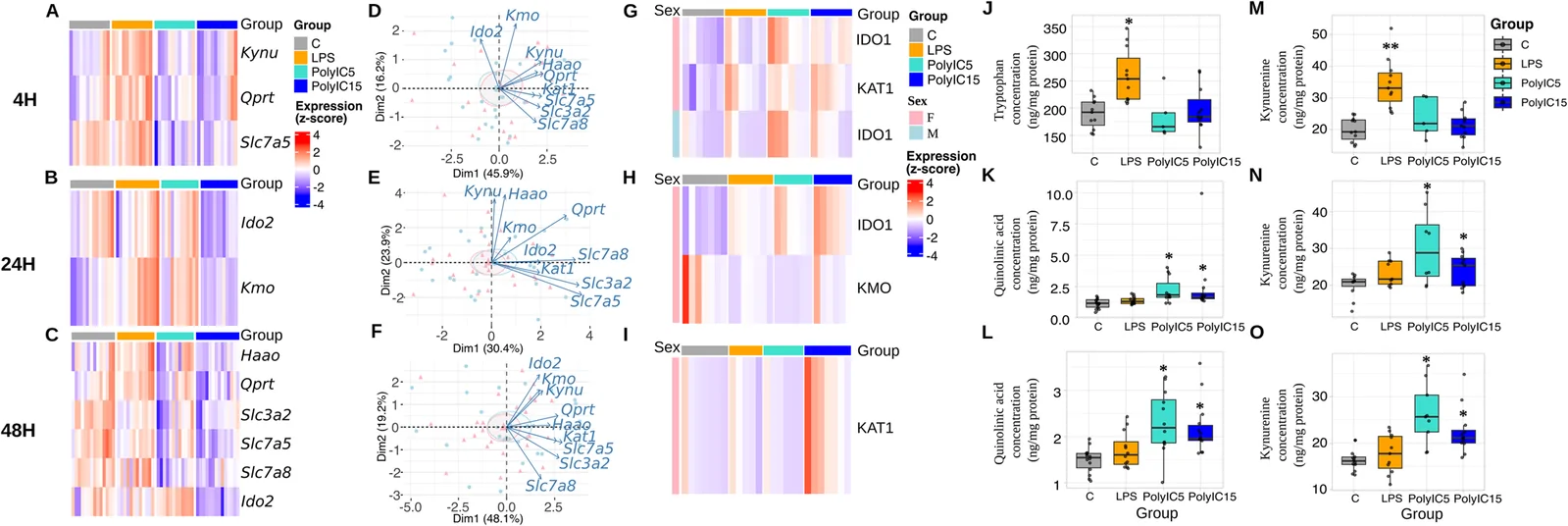
Placental kynurenine-pathway gene, protein, and metabolite responses following maternal immune activation. Heat maps (**A**—**C**): Normalized gene expression (z-scores) for transcripts that differed significantly from control within each time point. Genes include kynurenine-pathway enzymes (*Ido2*, *Kat1*, *Kmo*, *Kynu*, *Haao*, *Qprt*) and TRP transporters (*Slc7a5/*Lat1, *Slc7a8/*Lat2, *Slc3a2*). PCA biplots (**D**—**F**): Multivariate analysis of the same transcript datasets. Points represent sex-specific pooled placentas per litter (F = female, M = male); 95% confidence ellipses summarize within-group dispersion. Arrows indicate variable loadings, and axis labels show the percentage of variance explained by PC1 and PC2. Female and male clusters largely overlap within treatments, indicating no strong sex-dimorphic structure at the transcriptomic level. Western blot quantification (**G**–**I**): Protein levels of IDO1, KMO, and KAT in placental tissue at 4, 24, and 48 h. Heat maps showing protein with significant sex × treatment interactions at each time point, highlighting sex-dependent regulation of kynurenine-pathway components. Metabolite concentrations (**J**–**O**): Placental levels of tryptophan (TRP), kynurenine (KYN), kynurenic acid (KYNA), and quinolinic acid (QUIN). Data are presented for all samples combined, as no sex differences were detected, with only statistically significant differences displayed. *p < 0.05, ** *p* < 0.01 vs control

In contrast, the placenta exhibited a robust and time-dependent reprogramming of kynurenine-pathway activity at the transcriptional, protein, and metabolite levels (Fig. 4). At the gene expression level, MIA induced progressive and treatment-specific alterations, as illustrated by heat maps of transcripts significantly altered relative to controls at each time point (Fig. 4A–C; Supplementary Table 6). At 4 h, LPS exposure significantly increased *Qprt* and *Kynu* expression, whereas Poly I:C at both doses selectively reduced *Slc7a5* expression. By 24 h, Poly I:C 15 mg/kg was associated with reduced *Ido2* expression, while both LPS and Poly I:C 5 mg/kg induced *Kmo* upregulation. At 48 h, distinct treatment-specific patterns emerged: LPS tended to increase *Haao* expression and reduce *Slc3a2*; Poly I:C 5 mg/kg increased *Ido2* while downregulating all three transporter transcripts; and Poly I:C 15 mg/kg produced a coordinated suppression of *Ido2*, *Qprt*, and the transporter genes *Slc7a5*, *Slc7a8*, and *Slc3a2*. PCA of placental gene expression revealed overlapping clustering between sexes (Fig. 4D–F), and PERMANOVA confirmed the absence of global sex differences at the transcriptional level.

At the protein level, MIA induced selective and time-dependent modulation of placental kynurenine-pathway enzymes, with significant group effects observed for IDO1 at 4 h for Poly I:C 5 mg/kg and 24 h for both Poly I:C doses. For KAT, a trend toward increased expression was observed at 4 h, whereas at 48 h a significant global group effect was detected, although no pairwise differences remained significant after post hoc correction. KMO protein levels remained unchanged across all time points (Supplementary Fig. 7). Sex-stratified analysis revealed significant sex × treatment interactions; accordingly, the significant effects from these models are shown in Fig. 4G–I. In female placentas, IDO1 protein levels increased at 4 h following Poly I:C administration. A similar pattern persisted at 24 h, when IDO1 expression was elevated in response to LPS as well as both Poly I:C doses. KAT expression in females also increased at 4 h following Poly I:C exposure, remained unchanged at 24 h, and showed a trend toward increased expression at 48 h in the Poly I:C 15 mg/kg group. KMO protein levels did not differ significantly between treatment groups at any time point in females; however, a downward trend was observed at 24 h, consistent with a significant global group effect, although no pairwise differences remained significant after post hoc correction. In male-derived placentas, no statistically significant changes in IDO1, KMO, or KAT protein levels were detected at 4, 24, or 48 h following MIA. Although IDO1 levels showed an overall group effect at 4 h, post hoc comparisons did not reach statistical significance.

Metabolite profiling further demonstrated time dependent reconfiguration of placental kynurenine-pathway activity. At 4 h, KYN concentrations were significantly increased following LPS exposure (Fig. 4M), accompanied by an increase in TRP levels under the same condition (Fig. 4J), whereas KYNA and QUIN remained unchanged. By 24 h, KYN levels were elevated following Poly I:C exposure at both doses (Fig. 4N), while QUIN concentrations increased following Poly I:C 15 mg/kg (Fig. 4K), indicating differential engagement of downstream branches of the pathway. At 48 h, both KYN and QUIN concentrations were increased following Poly I:C administration at both doses (Fig. 4L and 4O), whereas KYNA and TRP remained unchanged. A sex × treatment interaction was formally tested for all placental metabolites and was non-significant in every case. Overall, these findings indicate that placental kynurenine-pathway metabolism undergoes progressive, dose-dependent reorganization following MIA, with early TRP/KYN mobilization giving way to sustained downstream QUIN accumulation by 48 h, independent of fetal sex. Collectively, these findings indicate that, in contrast to the modest and largely non–sex-dependent response observed in the decidua, the placenta exhibits a dynamic, time-dependent reprogramming of kynurenine-pathway activity following MIA, with sex-dependent effects emerging specifically at the protein level.

### Multivariate integration reveals stimulus-specific and time-dependent reorganization of kynurenine-pathway activity across the maternal–placental axis

**Fig. 5:**
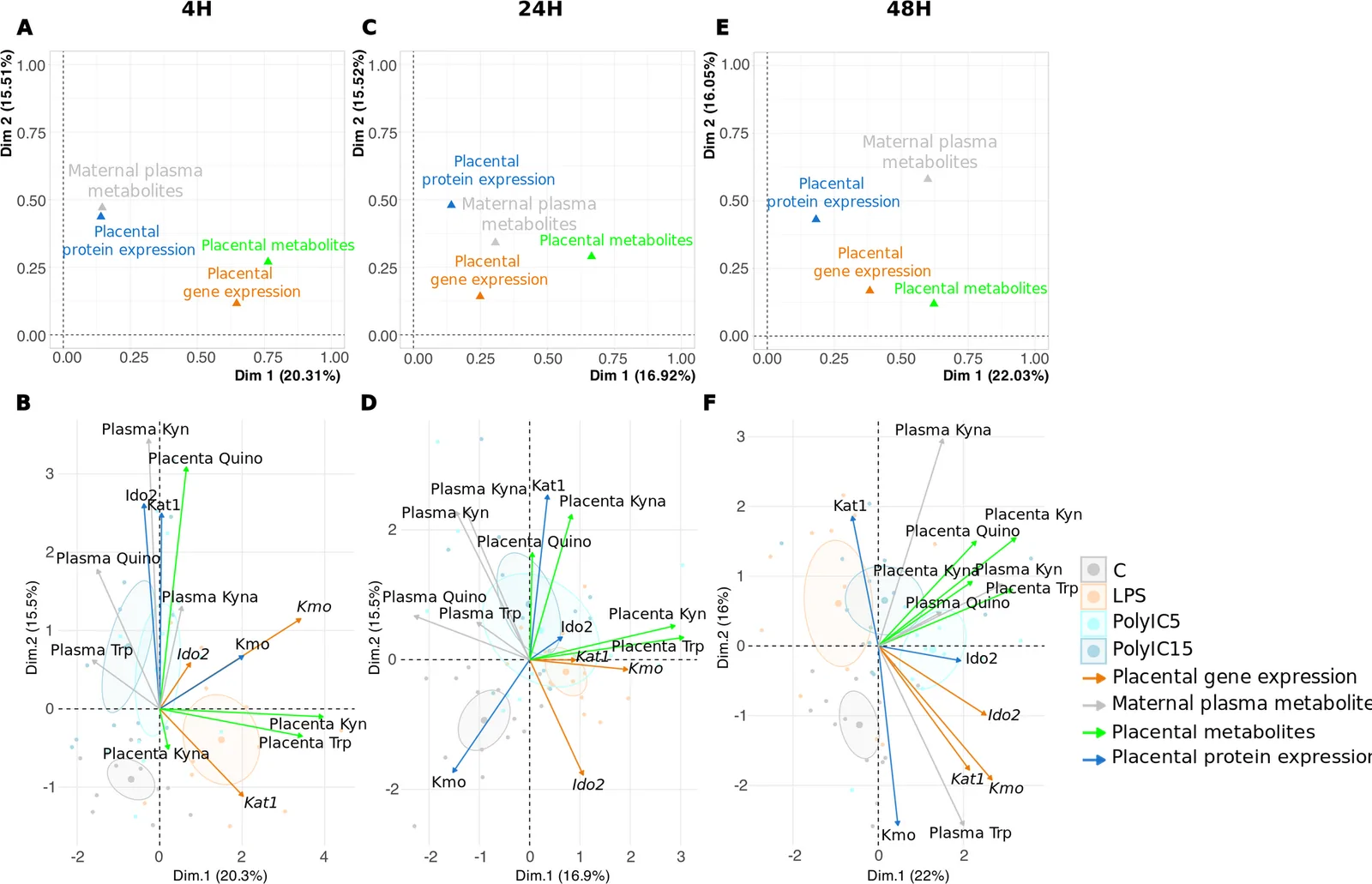
Multivariate integration of placental and maternal plasma kynurenine-pathway responses following MIA. MFA was performed at 4 h (**A**–**B**), 24 h (**C**–**D**), and 48 h (**E**–**F**) after MIA induced by Poly I:C (5 or 15 mg/kg) or LPS (1 mg/kg). Group representation plots (A, C, E) show the relative contribution of each data block to the first two MFA dimensions, including placental gene expression, placental protein expression, placental metabolites, and maternal plasma metabolites. Coordinates reflect the association of each block with Dim.1 and Dim.2, indicating their contribution to the global multivariate structure. Individual factor maps (B, D, F) display the distribution of samples and variable loadings. Points represent individual samples colored by treatment group (Control, LPS, Poly I:C 5 mg/kg, Poly I:C 15 mg/kg), and ellipses indicate 95% confidence intervals for each group. Arrows represent variables, including placental kynurenine-pathway gene expression (*Ido2*, *Kmo*, *Kat1*), protein levels (IDO1, KMO, KAT), and metabolite concentrations in placenta and maternal plasma (TRP, KYN, KYNA, QUIN). The direction and length of arrows indicate the contribution and correlation of each variable with the principal dimensions

To integrate transcriptional, protein, and metabolite responses across placental datasets and maternal plasma, MFA was performed at 4, 24, and 48 h following MIA (Fig. 5). Group representation analysis showed that placental metabolites were the dominant contributors to Dim.1 at all time points (4 h: 45.0%; 24 h: 48.6%; 48 h: 34.8%), whereas maternal plasma metabolites and placental protein expression contributed more prominently to Dim.2 (maternal metabolites: 36.3%, 27.3%, 40.7%; proteins: 33.9%, 38.1%, 35.5%).

At 4 h, placental gene expression and metabolites profiles were closely aligned, consistent with coordinated early transcriptional and metabolic activation. This concordance was markedly reduced at 24 h (RV = 0.071) and remained low at 48 h (RV = 0.086). In contrast, placental protein expression showed increasing structural similarity with the global MFA configuration over time (RV = 0.508, 0.566, and 0.591 at 4, 24, and 48 h, respectively). Notably, placental protein expression remained weakly correlated with both placental gene expression and maternal metabolites throughout the time course (Fig. 5A, C, and E).

Analysis of variable contributions further revealed distinct stimulus-specific temporal dynamics. These patterns were confirmed by PERMANOVA, which demonstrated a significant effect of treatment group at all time points (4 h: F = 4.84, R^2^ = 0.241, *p* = 0.001; 24 h: F = 4.49, R^2^ = 0.215, *p* = 0.001; 48 h: F = 3.91, R^2^ = 0.194, *p* = 0.001). LPS induced a rapid and predominantly placental-localized response at 4 h, characterized by coordinated variation in placental gene expression (*Kmo*, Dim.1: r = + 0.780) and early metabolites, including TRP and KYN, with the greatest multivariate displacement from controls (Ed = 2.66, *p* = 0.001). This response diminished by 24 h (Ed = 1.88, *p* = 0.001) and was further attenuated at 48 h (Ed = 1.52, *p* = 0.002).

In contrast, Poly I:C elicited a more systemic and temporally delayed response. At 4 h, variation was driven primarily by maternal plasma metabolites and placental protein expression, with limited contribution from placental metabolites (Poly I:C 5 mg/kg: Ed = 2.08; Poly I:C 15 mg/kg: Ed = 1.31; *p* = 0.001 for both). From 24 h onward, placental metabolites, particularly KYN (Dim.1: r = + 0.724, *p* < 0.001) and QUIN, became the dominant contributors to group separation (Poly I:C 5 mg/kg: Ed = 2.06; Poly I:C 15 mg/kg: Ed = 2.74; *p* = 0.001 for both), indicating progressive activation of downstream kynurenine-pathway metabolism. At 48 h, Poly I:C 5 mg/kg maintained a comparable displacement (Ed = 2.01, *p* = 0.002), whereas Poly I:C 15 mg/kg showed a reduced but still significant separation (Ed = 1.70, adjusted p = 0.002).

As a whole, these findings demonstrate that MIA induces stimulus-specific and time-dependent reorganization of kynurenine-pathway activity, characterized by rapid, transient placental-localized responses to LPS and delayed, sustained metabolic reprogramming following Poly I:C exposure (Fig. 5B, D, and F).

### Fetal brain shows pronounced, time- and sex-dependent transcriptional changes in kynurenine-pathway genes after maternal immune activation

**Fig. 6:**
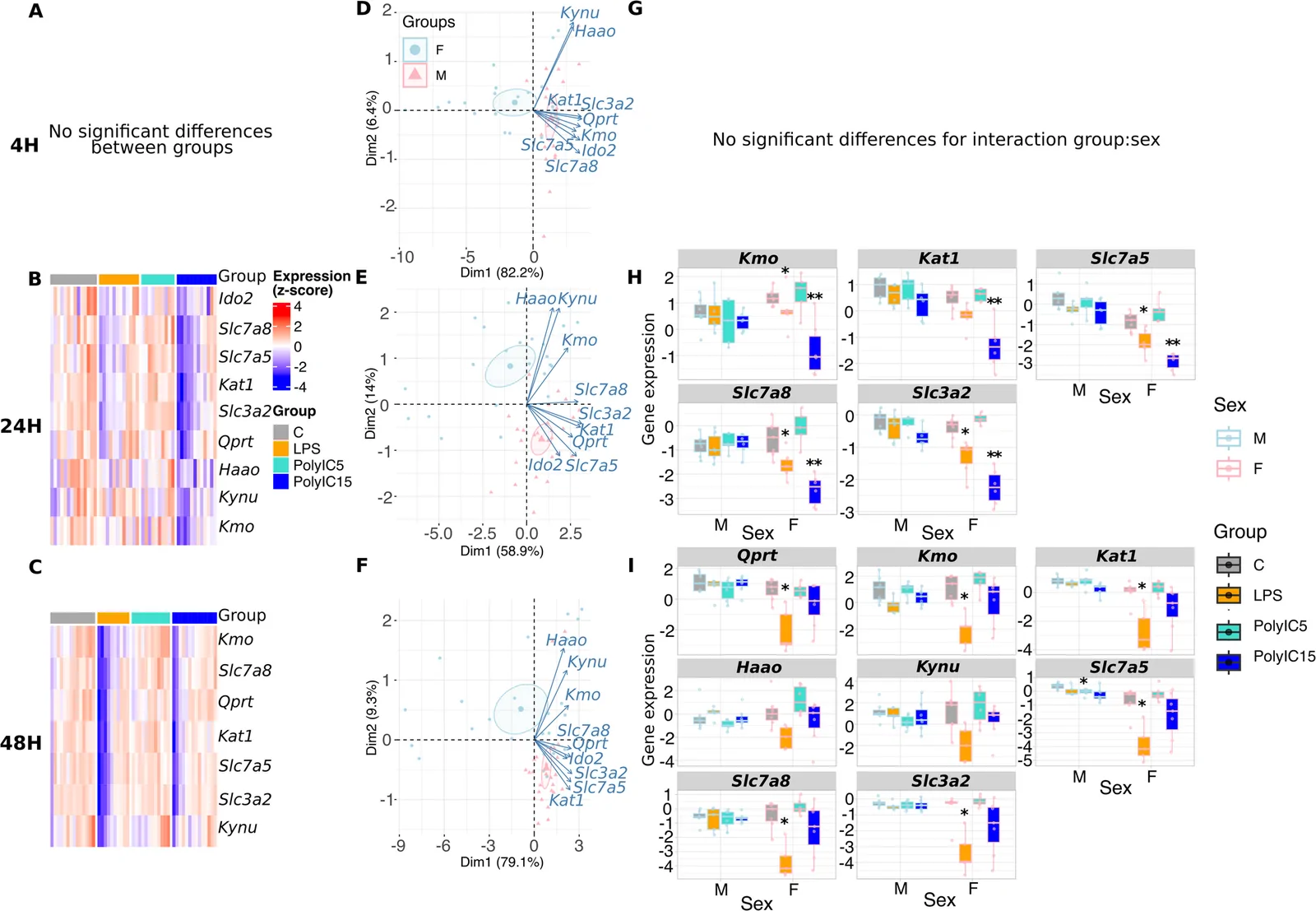
Transcriptional remodeling of kynurenine-pathway genes in the fetal brain following maternal immune activation. **A**–**C** Heat maps showing normalized gene expression (z-scores) of kynurenine-pathway enzymes (*Ido2, Kmo, Kynu, Haao, Qprt, Kat1*) and amino acid transporters (*Slc7a5/Lat1, Slc7a8/Lat2, Slc3a2*) that differed significantly from control in fetal brain tissue at 4 h (**A**), 24 h (**B**), and 48 h (**C**) after MIA. At 4 h, no significant differences between treatment groups were detected. Group colour bars indicate Control, LPS (1 mg/kg), Poly I:C 5 mg/kg, and Poly I:C 15 mg/kg. (**D**–**F**) PCA biplots of fetal brain transcript expression at 4 h, 24 h, and 48 h. Points represent pooled fetal brain samples by sex (female, F; male, M), with 95% confidence ellipses shown for each sex within treatment groups. Arrows indicate variable loadings, with direction and length reflecting relative contributions to PC1 and PC2. Axis labels report the percentage of variance explained. Sex-dependent separation within treatments emerges at 24 h and becomes more pronounced at 48 h. (**G**) Summary indicating no significant sex × treatment interaction effects at 4 h. (**H**–**I**) Boxplots depicting transcripts with significant sex × treatment interaction effects at 24 h (**H**) and 48 h (**I**). Data are shown as individual values with group medians and interquartile ranges. Statistical analysis is described in methods. Asterisks denote significant differences relative to sex-matched controls (*p < 0.05, **p < 0.01)

Building on the fetal brain PCA findings described above (Fig. 3), which showed significant multivariate separation emerging at 24 and 48 h, we next assessed treatment-induced changes in individual kynurenine-pathway and System L transporter genes in the fetal brain relative to controls. Heat map analysis revealed broad transcriptional shifts relative to control that intensified over time (Fig. 6). No group differences were detected at 4 h. By 24 h, nine transcripts were differentially expressed. Poly I:C 15 mg/kg induced coordinated downregulation of *Ido2*, *Kat1*, *Kmo*, *Kynu*, *Qprt*, *Haao*, *Slc7a5*, *Slc7a8*, and *Slc3a2,* while LPS selectively reduced *Slc3a2*. At 48 h, the LPS effect intensified, with reduced expression of seven genes (*Kat1, Kmo, Qprt, Slc7a5, Slc7a8, Slc3a2,* and *Kynu*) (Fig. 6 A-C; Supplementary Table 7). PCA of the same dataset, with samples grouped by sex, revealed sex-dependent separation that emerged at 24 h and became pronounced at 48 h (Fig. 6D–F). Formal sex × treatment interaction testing detected no significant interaction at 4 h (Fig. 6G), whereas specific kynurenine-pathway and transporter genes showed significant interactions at 24 and 48 h (Fig. 6H, I). At 24 h, female fetal brains exhibited selective downregulation of *Kmo*, *Slc7a5*, *Slc7a8*, and *Slc3a2* following LPS and Poly I:C 15 mg/kg exposure, whereas *Kat1* expression was reduced exclusively in females treated with Poly I:C 15 mg/kg. By 48 h, this pattern became more pronounced, with LPS-treated females showing reduced expression of *Qprt*, *Kmo*, *Kat1*, *Slc7a5*, *Slc7a8*, and *Slc3a2*. In addition, *Haao* and *Kynu* expression showed a downward trend under LPS treatment in females. No comparable transcriptional changes were detected in male fetal brains at either time point, with the exception of a downregulation of *Slc7a5* in males treated with Poly I:C 5 mg/kg at 48 h. Altogether, these findings, in contrast to the minimal sexual dimorphism observed in placenta and decidua, identify the fetal brain as a site of sex-specific transcriptional vulnerability to MIA.

To directly test the coordination implied by the placenta–fetal brain axis, we examined whether transcript levels of the six kynurenine-pathway genes measured in both compartments (*Ido2*, *Kmo*, *Kynu*, *Haao*, *Qprt*, and *Kat1*) co-varied within litters, independent of treatment (Supplementary Table 8). After adjustment for treatment group, time point, and fetal sex, placental and fetal-brain transcript levels correlated for two of the six genes: *Kmo* showed a positive within-litter correlation (partial Spearman ρ = 0.27, q = 0.0045) and *Kat1* an inverse correlation (ρ = − 0.26, q = 0.0045); the remaining four genes showed no significant placenta–brain coupling. This pattern held in the same direction when re-estimated within each treatment group, and the placenta × treatment interaction was non-significant for all six genes, indicating that the strength of placenta–brain coupling did not differ detectably between treatments.

## Discussion

**Fig. 7:**
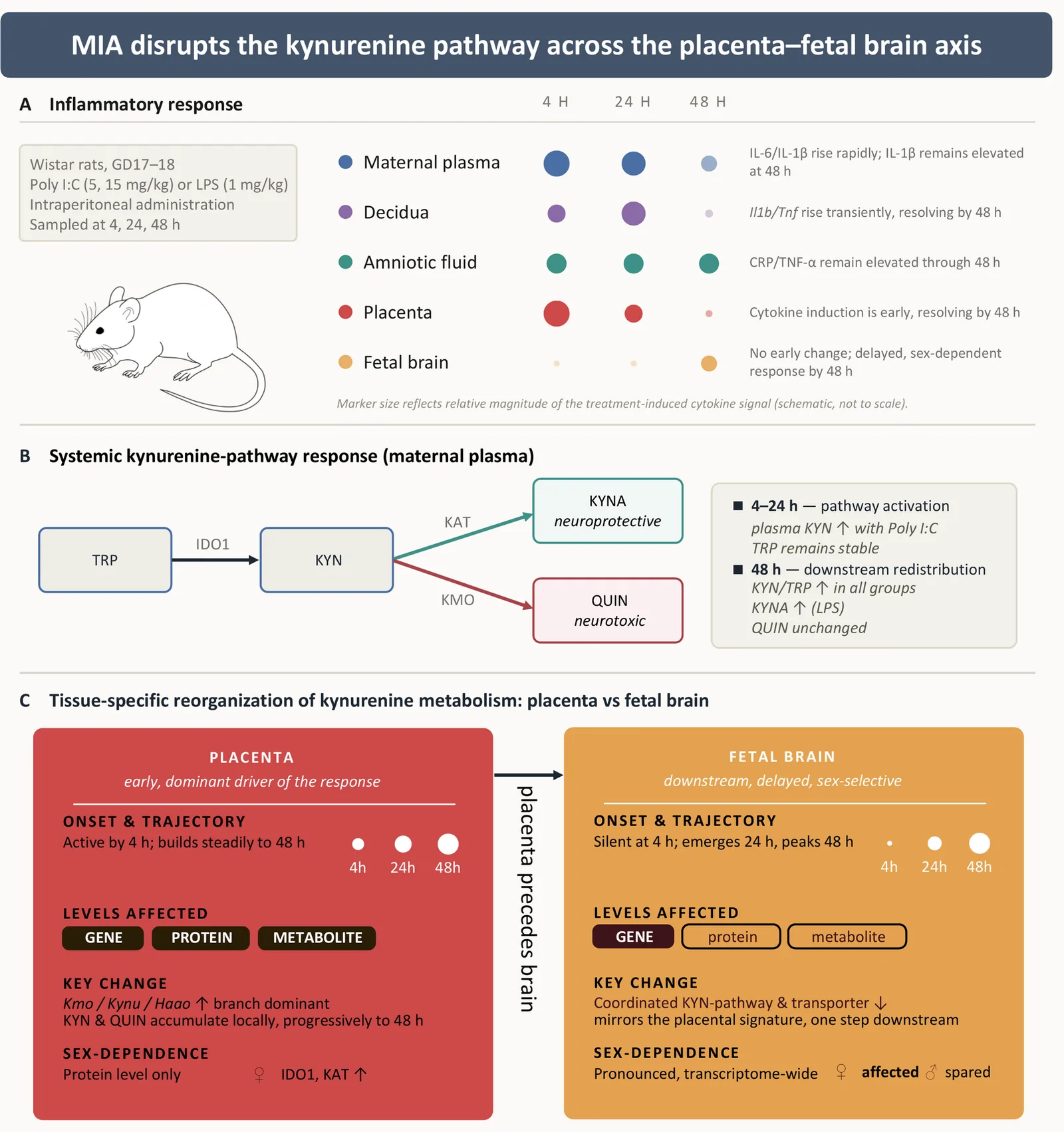
Graphical summary of MIA-induced remodeling of the kynurenine pathway across the placenta–fetal brain axis. **A** Schematic overview of the inflammatory response across five compartments (maternal plasma, decidua, amniotic fluid, placenta, fetal brain) at 4, 24, and 48 h following the final maternal injection of Poly I:C (5 or 15 mg/kg) or LPS (1 mg/kg). Marker size reflects the relative magnitude of the treatment-induced cytokine signal in each compartment and timepoint (schematic, not to scale). **B** Schematic of the systemic kynurenine pathway in maternal plasma, illustrating the conversion of tryptophan (TRP) to kynurenine (KYN) via indoleamine 2,3-dioxygenase 1 (IDO1), and the downstream branching toward the neuroprotective metabolite kynurenic acid (KYNA, via kynurenine aminotransferase, KAT) or the neurotoxic metabolite quinolinic acid (QUIN, via kynurenine 3-monooxygenase, KMO). Temporal annotations summarize the direction of metabolite changes at 4–24 h (pathway activation) and at 48 h (downstream redistribution), as reported in Table 1. **C** Comparative schematic of tissue-specific kynurenine-pathway reorganization in the placenta and fetal brain, summarizing the onset and trajectory of the response, the biological levels affected (gene, protein, metabolite), the dominant direction of change, and the pattern of sex-dependence in each tissue, as detailed in the Results and Discussion

Our findings identify the placenta as an early and prominent site of immunometabolic remodeling following MIA, with pronounced restructuring of TRP metabolism along the KYN pathway. This response was temporally organized and stimulus-specific, with rapid, transient placental activation following LPS and delayed, sustained metabolic reprogramming in response to Poly I:C. Although placental transcriptional responses were largely non–sex-dimorphic, sex-dependent differences emerged at the protein levels and became more pronounced in the fetal brain, where transcriptional changes were delayed and exhibited marked female-biased sensitivity. Together, these findings are consistent with a model in which placental responses to inflammation are associated with altered fetal exposure to neuroactive metabolites, with potential implications for neurodevelopmental trajectories in a time- and sex-dependent manner (Fig. 7).

To model distinct inflammatory contexts, we employed Poly I:C and LPS to recapitulate viral- and bacterial-like MIA in late gestation. These stimuli engage different innate immune pathways and cytokine programs (Vercammen et al. 2008; Dauphinee and Karsan 2006), providing a framework to assess how qualitatively distinct inflammatory signals modulate TRP metabolism within the placenta–fetal brain axis.

The successful induction of maternal inflammation was confirmed by elevated concentrations of IL-6 at 4 h and IL-1β at 24 h in maternal plasma across all treatment groups, together with increased levels of CRP and TNF-α in amniotic fluid, and increased cytokine transcript expression in placental and decidual tissue. As expected, Poly I:C and LPS triggered a rapid rise in circulating cytokines, consistent with previous reports of systemic immune activation following viral- and bacterial-like stimuli (Arrode-Brusés and Brusés 2012; Bao et al. 2022). Poly I:C elicited a broader and more sustained cytokine response than LPS, consistent with interferon-mediated activation characteristic of viral-like immune stimulation. Analysis of amniotic fluid further demonstrated that inflammatory signaling reached the maternal–fetal interface, where cytokine profiles exhibited clear sex-dependent differences. Female-derived samples showed earlier and broader cytokine activation, consistent with an increased innate immune responsiveness, whereas males displayed a more restricted response. These differences in CRP, IL-1β, TNF-α, and IL-6 suggest intrinsic sex-based variation in inflammatory regulation at the maternal–fetal interface, consistent with our own finding of a female-specific increase in decidual *Il1b* expression following LPS exposure. A similar pattern of heightened inflammatory gene expression in female placentas following MIA has been reported by Biggar et al., supporting sex-dependent immune responsiveness across compartments of the maternal–fetal interface (Biggar et al. 2025). Together, these findings indicate intrinsic sex-dependent differences in immune regulation at the maternal–fetal interface, potentially contributing to the sex-biased susceptibility to neurodevelopmental disorders (Yang et al. 2024). Dysregulated pro-inflammatory cytokine signaling during critical windows of brain development can disrupt neurogenesis, synaptic pruning, and axonal maturation by activating microglia and promoting excitotoxic and oxidative stress, thereby increasing susceptibility to persistent neurodevelopmental impairment (Jiang et al. 2018; Ratnayake et al. 2013). IL-6 signaling, in particular, has been implicated as a causal mediator of altered cortical maturation and synaptic organization (Smith et al. 2007). The progressive elevation of CRP within amniotic fluid is consistent with clinical observations in preterm birth cohorts, where local inflammatory mediators predict placental metabolic dysregulation, including altered TRP metabolism and downstream KYN pathway remodeling (Karahoda et al. 2021).

MIA altered fetal growth in a stimulus-specific and time-dependent manner, consistent with reports that immune challenge influences fetal growth and early development in rat MIA models (Sandovici et al. 2012; Murray et al. 2019). The early reduction in fetal weight observed across all treatment groups at 4 h corresponds to approximately 28 h after the first injection. This timing suggests the reduction likely reflects the acute maternal sickness response and associated decrease in food intake (Hopwood et al. 2009), although its magnitude is unlikely to be fully explained by these factors alone. From 24 h onward, growth trajectories diverged in a stimulus-specific manner. Poly I:C was associated with reduced fetal weight that persisted at both doses through 48 h, paralleling its broader and more sustained inflammatory and metabolic signature. LPS-exposed fetuses, by contrast, became heavier than controls over the same period. Analysis of pregnancy outcome showed low post-implantation loss in the LPS group, indicating that the heavier phenotype is unlikely to reflect selective loss of growth-compromised fetuses and is better explained by the transient nature of the bacterial-like response. Litter size was also included as a factor in all biometric models, confirming that the observed differences are not attributable to litter size alone. In contrast, the Poly I:C 5 mg/kg group showed significantly higher post-implantation loss at 48 h, reflecting substantial variability between litters rather than a uniform increase across the group. This pattern is consistent with the variable fetal loss reported for high molecular weight Poly I:C (Mueller et al. 2019), and the fetal weight pattern in this group is therefore interpreted with greater caution. The increased brain-to-fetal weight ratio observed at 48 h in this group is consistent with an asymmetric, brain-sparing pattern of growth restriction, a recognized signature of placental insufficiency in models of intrauterine growth restriction (Felicioni et al. 2020; Fleiss et al. 2019). At the placental level, downregulation of System L amino acid transporter transcripts (Slc7a5/LAT1, Slc7a8/LAT2, and Slc3a2) at later time points should be interpreted as a transcriptional signature rather than direct evidence of altered transporter protein abundance or transport activity. Nevertheless, because these transporters mediate the exchange of large neutral amino acids, including TRP, their downregulation may reflect reduced substrate availability at the maternal–fetal interface, potentially contributing to altered fetal growth and downstream metabolic signaling. This interpretation is consistent with previous reports that inflammatory cytokines, including IL-6 and TNF-α, disrupt placental amino acid transport systems, including LAT and SNAT transporters, in both rodent MIA models and human cohorts (Francis et al. 2025; Kowash et al. 2022; McColl et al. 2023; Shimada et al. 2024).

Given that TRP is primarily metabolized through the KYN pathway (Badawy 2015), alterations in its placental availability are expected to directly impact downstream metabolic flux. Metabolites generated through the KYN pathway exhibit both pro-oxidant and antioxidant properties and play pivotal roles in maintaining pregnancy, contributing to immune tolerance, regulation of placental and maternal vascular tone, and neuroprotection (Zundert et al. 2022). Maternal inflammation has been shown to disturb TRP metabolism, thereby altering flux through the KYN pathway (Abad et al. 2024; Williams et al. 2017). In our study, MIA enhanced systemic TRP catabolism through the KYN pathway, as reflected by elevated maternal plasma KYN levels and an increased KYN/TRP ratio following both LPS and Poly I:C exposure. This pattern indicates accelerated TRP degradation under inflammatory conditions, consistent with previous reports using bacterial (LPS) or viral (Poly I:C) mimetics (Larsson et al. 2016; Yang et al. 2025). Notably, maternal levels of KYNA were also elevated following LPS exposure, consistent with previous observations in mice, suggesting a compensatory antioxidant and anti-inflammatory response within the maternal compartment (Michaudel et al. 2023).

A key consequence of the inflammatory environment was the reprogramming of placental TRP metabolism toward the KYN pathway. This was most clearly reflected at the protein level, where IDO1 showed a sustained increase at 4 and 24 h, consistent with the well-established evidence that pro-inflammatory cytokines upregulate IDO1 expression and enhance KYN production (Murthy et al. 2021; Yamada et al. 2009). This finding parallels observations in human placental explants exposed to viral or bacterial mimetics, where inflammation similarly induces IDO1 activation and KYN synthesis (Abad et al. 2024). In contrast, the *Ido2* transcript, which is not known to be cytokine-inducible, changed in a mixed, dose- and time-dependent manner rather than showing a coordinated inflammatory signature. We therefore interpret the *Ido2* transcript and IDO1 protein findings separately rather than as evidence of the same regulatory event. Because *Ido1* transcript is expressed at very low levels in the rat placenta relative to *Ido2* (Abad et al. 2020), the detection of an inflammation-responsive IDO1 protein signal in this tissue represents a discordance between transcript and protein levels. This discordance may reflect differences in mRNA/protein stability, post-transcriptional regulation, or temporal offsets between transcription and translation (Maier et al. 2009). Whether IDO2 is similarly regulated at the protein level in response to inflammation remains unknown, as this has not, to our knowledge, been characterized in placental tissues, in contrast to the well-established cytokine-inducibility of IDO1 (Wu et al. 2018). Beyond *Ido2*, both Poly I:C and LPS altered the expression of several enzymes, including *Kmo*, *Kynu*, and *Qprt*, with effects becoming more pronounced over time. Several downstream metabolites are generated from KYN, including QUIN, an NMDA receptor agonist, and KYNA, formed through an alternative metabolic branch, which functions as an antagonist of both NMDA and α7 nicotinic acetylcholine receptors. The upregulation of *Kmo* and *Kynu* indicates enhanced KYN catabolism through the KMO branch, promoting the generation of the neurotoxic metabolite QUIN. In contrast, *Qprt*, despite a transient early increase under LPS, was downregulated under Poly I:C 15 mg/kg by 48 h, suggesting reduced conversion of QUIN into its downstream products, potentially leading to its accumulation within placental tissue, a mechanism similarly implicated in elevated placental QUIN levels and maternal depressive symptoms during pregnancy (Sha et al. 2024).

Interestingly, downstream enzyme expression displayed sex-specific patterns at the protein level. In female placentas, KAT protein levels increased at 4 h following Poly I:C exposure, with a similar trend at 48 h, suggesting a preferential shift toward the neuroprotective branch of the KYN pathway. In contrast, no significant changes in KAT or KMO protein levels were detected in male-derived placentas.

At the metabolite level, placental KYN and QUIN concentrations increased markedly following Poly I:C exposure at 24 and 48 h, confirming a treatment-driven shift toward the neurotoxic branch of the KYN pathway that may increase fetal exposure to neurotoxic metabolites. This pattern aligns with clinical metabolomic data showing increased KYN and QUIN levels in inflamed human placentas (Cifkova et al. 2024; Abad et al. 2024). Formal testing of the sex × treatment interaction was non-significant for all placental metabolites at every time point. Descriptively, KYNA concentrations appeared relatively higher in females and QUIN relatively higher in males. However, because the formal interaction was not significant, we do not interpret this apparent divergence as evidence of a true sex difference. Given the female-specific KAT protein increase described above, we consider this descriptive pattern a hypothesis-generating observation warranting further testing in an adequately powered, dedicated cohort.

This hypothesis is consistent with, and motivated by, findings from other MIA models: a recent study by Sun et al. (Sun et al. 2025) demonstrated that inflammatory signaling during pregnancy reprograms placental and fetal brain metabolism toward the KMO–QUIN branch, leading to increased production of the neurotoxic metabolite QUIN exclusively in male offspring. Similarly, Hasegawa et al. (Hasegawa et al. 2024) showed that MIA enhances placental KMO activity and 3-HK production, driving oxidative stress, cortical abnormalities, and behavioral deficits in adult male offspring. Importantly, both pharmacological inhibition and genetic deletion of KMO prevented these alterations, identifying placental KMO as a potential therapeutic target for mitigating MIA-related neuropsychiatric risk. Should our hypothesis-generating observation be confirmed in an adequately powered study, differential routing of KYN toward neuroprotective (KYNA) or neurotoxic (QUIN) metabolites could represent one mechanism by which placental KYN metabolism contributes to sex-biased vulnerability to neuropsychiatric disorders.

Although transcriptional alterations were detected in the decidua, they were comparatively modest and showed no sex-specific pattern. This suggests that the decidua may function as an immunological buffer, attenuating maternal cytokine transmission while only partially constraining downstream metabolic adaptations. Notably, this observation contrasts with previous reports describing the decidua as an immunologically active compartment (Lv et al. 2025; Mor et al. 2017) with significant IDO expression (Miwa et al. 2005; Rango et al. 2007) and the capacity to upregulate IDO1 in response to inflammatory stimuli (Wang et al. 2022). Despite this, we did not observe corresponding changes in IDO1, KMO, or KAT at the protein level, nor evidence of substantial kynurenine-pathway reprogramming in this tissue. These findings indicate that, under the conditions tested, the decidua plays a limited role in TRP–KYN metabolic regulation compared with the placenta, which emerges as the principal site of inflammation-driven metabolic reprogramming.

In contrast, fetal brain gene expression revealed pronounced suppression of metabolic enzymes and transporters at 24–48 h. Downregulation of *Qprt*, which converts QUIN into nicotinate mononucleotide, implies reduced detoxification capacity, potentially exacerbating QUIN accumulation. Likewise, the observed reduction in *Slc7a5* (LAT1) and *Slc7a8* (LAT2) transcripts could, if accompanied by corresponding changes in protein abundance and activity, limit the transport of large neutral amino acids into the fetal brain. This could, in turn reduce substrate competition at NMDA receptors, thereby increasing sensitivity to neurotoxic metabolites such as QUIN. Functional impairment of System L transport has been demonstrated in MIA models and under inflammatory stress, where reduced LAT1/LAT2 activity restricts amino acid delivery to the brain and contributes to neurodevelopmental vulnerability (Kowash et al. 2022; Gyawali et al. 2020; Kahlhofer and Teis 2023). These findings support the view that the fetal brain functions as an indirect metabolic target of placental inflammation rather than a primary source of cytokine induction (Pocivavsek et al. 2024), underscoring the critical interdependence between placental function and fetal brain development within the placenta–brain axis (Rosenfeld 2021).

Beyond these shared transcriptional targets, the fetal brain kynurenine-pathway response was itself markedly sex-dependent. Female-derived samples showed coordinated downregulation of *Kmo*, *Kat1*, *Qprt*, and the System L transporter genes following LPS and Poly I:C exposure, while no comparable changes were detected in males. Several of these enzymes govern the relative production of QUIN, an NMDA receptor agonist, and KYNA, an NMDA receptor antagonist. Because NMDA receptor signaling is a central regulator of neurogenesis, progenitor migration, and synapse formation (Chakraborty et al. 2017; Paoletti et al. 2013), a sex-dependent shift in their expression could plausibly influence male and female fetal brain development differently. We note, however, that *Kmo* and *Kat1* were suppressed together in females, rather than in the divergent pattern that would indicate a clean shift toward either the neurotoxic or neuroprotective branch. The functional consequence of this broader, coordinated suppression for the KYNA/QUIN balance in the female fetal brain therefore remains uncertain and cannot be resolved from transcript-level data alone.

This female-specific pattern parallels findings in the adult human brain, where KYNA is specifically reduced in the anterior cingulate cortex of women with major depressive disorder, but not men, a difference linked to *KAT* pathway activity (Brown et al. 2024). Sex-dependent remodeling of the fetal brain kynurenine–NMDA axis following MIA has also been reported in other rodent models, although the affected sex has varied with species, gestational stimulus, and timing (Santana-Coelho et al. 2026).

Epidemiological and experimental evidence consistently indicates that males are more vulnerable to early-onset neurodevelopmental conditions such as autism spectrum disorder, ADHD, and schizophrenia, whereas females exhibit a higher lifetime risk of depression, anxiety, and stress-related disorders, particularly during adolescence and reproductive years. These disparities reflect sex-specific immune, hormonal, and stress-response mechanisms that shape neurodevelopmental trajectories and disease vulnerability (Yang et al. 2024; Ardalan et al. 2019; Klein and Flanagan 2016).

Experimental manipulation of brain KYNA levels in rodents has been shown to induce cognitive deficits, including impairments in contextual learning and memory, whereas increased QUIN concentrations have been implicated in neuropsychiatric conditions such as schizophrenia (Blanco Ayala et al. 2021; Heyes et al. 1992; Anderson et al. 2013). The female-biased suppression of KYN-pathway enzymes observed here, considered alongside this literature, raises the hypothesis that placental and fetal brain kynurenine metabolism may contribute to sex-specific programming of neurodevelopmental and psychiatric risk; confirming this will require functional and behavioral validation.

A further limitation is that fetal brain tissue was analyzed as a homogenate, pooling all cell types and anatomical regions. Because QUIN is generated predominantly by microglia (Guillemin 2012) and KYNA predominantly by astrocytes (Guillemin et al. 2001), bulk tissue analysis cannot distinguish whether the observed sex-dependent transcriptional changes reflect shifts in cell-type composition, cell-type-specific expression, or region-specific effects. Cell-type- and region-resolved analyses will be needed to clarify which populations and brain areas drive this sex-dependent vulnerability.

PCA revealed that the placenta exhibited the earliest and most robust transcriptional response, whereas changes in the fetal brain emerged later, indicating a temporally delayed adaptation. This pattern is consistent with a sequential model in which maternal cytokine signaling reprograms placental metabolic pathways, potentially altering the metabolite environment available to the fetus and thereby contributing to the subsequent changes observed in fetal brain gene expression. In support of this model, placental and fetal-brain transcript levels of *Kmo* and *Kat1* correlated within litters even after adjusting for treatment group, time point, and fetal sex. This indicates that these two compartments co-vary beyond what would be expected if both simply responded independently to the same maternal stimulus. This within-litter coupling remains, however, an association: it cannot establish the direction of the relationship, nor fully exclude a shared litter-level maternal factor influencing both tissues. Only an intervention directly blocking the placental response could demonstrate a coordinating mechanism. Such temporal coupling between placental and neural responses aligns with the Developmental Origins of Health and Disease (DoHaD) framework, which posits that transient gestational perturbations can induce lasting neurodevelopmental alterations (Barker 1998).

A major strength of this study is the time-resolved sampling at 4, 24, and 48 h after the final Poly I:C or LPS injection on GD18. Because MIA-induced metabolic and inflammatory changes are transient, this design enabled the detection of dynamic molecular responses that single time-point studies may overlook. Several limitations merit consideration. First, late-gestation exposure may not capture earlier windows of vulnerability during corticogenesis. Second, the use of pooled tissues provides population-level insight but may obscure interindividual variability inherent to developmental programming. Third, metabolite quantification was limited to placental tissue; direct assessment in the fetal brain would better define central nervous system exposure gradients. Nevertheless, this model recapitulates key hallmarks observed in human cohorts, including inflammatory induction of IDO1, a metabolic shift toward QUIN, and suppression of amino acid transporters expression. The convergence across species and experimental platforms underscores the translational relevance of placental kynurenine-axis modulation as a candidate mechanistic contributor to neurodevelopmental risk.

## Conclusions

MIA during late gestation elicits structured immunometabolic remodeling within the placenta–fetal brain axis. This process involves transient but coordinated perturbations in TRP metabolism, characterized by cytokine-driven induction of placental KYN enzymes and metabolite profiles with downstream alterations in fetal brain gene expression. Distinct time-, dose-, and sex-dependent patterns highlight intrinsic developmental vulnerabilities, particularly in metabolic nodes regulating glutamatergic tone. Following MIA, the placenta showed early and pronounced remodeling of the kynurenine pathway that preceded and was statistically associated with sex-specific changes in the fetal brain. This temporal and associative pattern identifies placental KYN metabolism as a candidate mediator linking maternal inflammation to differential neurodevelopmental vulnerability.

## Supplementary Information

Supplementary Material 1.

Supplementary Material 2.

## Data Availability

The data used and analyzed during the current study are available in the Zenodo repository, https://doi.org/10.5281/zenodo.21886962.

## Abbreviations

ADHD: Attention deficit/hyperactivity disorder
ASD: Autism spectrum disorder
CRP: C-reactive protein
GD: Gestational day
IDO: Indoleamine 2,3-dioxygenase
IFN-γ: Interferon gamma
IL: Interleukin
KAT: Kynurenine aminotransferase
KMO: Kynurenine monooxygenase
KYN: Kynurenine
KYNA: Kynurenic acid
LAT: Large neutral amino acid transporter
LPS: Lipopolysaccharide
MFA: Multiple factor analysis
MIA: Maternal immune activation
NMDA: N-methyl-D-aspartate
PCA: Principal component analysis
PERMANOVA: Permutational multivariate analysis of variance
Poly I:C: Polyinosinic:polycytidylic acid
QUIN: Quinolinic acid
TRP: Tryptophan
TNF-α: Tumor necrosis factor alpha
