# Supplementary material for: Maternal immune activation disrupts the kynurenine pathway of tryptophan metabolism in the placenta–fetal brain axis in rats
Source: Mol Med. 2026 Aug 7;32:214. doi: 10.1186/s10020-026-01587-1 (PMC13749810; doi:10.1186/s10020-026-01587-1)
Supplement: Supplementary file 1 [file 10020_2026_1587_MOESM1_ESM.docx]

**Maternal immune activation disrupts the kynurenine pathway of tryptophan metabolism in the placenta–fetal brain axis in rats**

Supplementary information

**Supplementary Figure 1. Sex-dependent cytokine profiles in decidua following maternal immune activation.** *Tnf* (A–C), *Il1b* (D–F), *Il6* (G-I) and *Ifng* (J-L) expression in decidua at 4 h, 24 h, and 48 h following maternal immune activation. Data are presented as boxplots stratified by sex (M, male; F, female) and treatment group (C, control; LPS; Poly I:C 5 mg/kg; Poly I:C 15 mg/kg). Statistical significance is indicated by asterisks (**p* < 0.05) relative to sex-matched controls. Points represent individual samples; boxes indicate median and interquartile range.

**Supplementary Figure 2. Sex-dependent cytokine profiles in amniotic fluid following maternal immune activation.** TNF-α (A–C), IL-6 (D–F), and CRP (G–I) concentrations in amniotic fluid at 4 h, 24 h, and 48 h following maternal immune activation. Data are presented as boxplots stratified by sex (M, male; F, female) and treatment group (C, control; LPS; Poly I:C 5 mg/kg; Poly I:C 15 mg/kg). Statistical significance is indicated by asterisks (**p* < 0.05, ***p* < 0.01, ****p* < 0.001) relative to sex-matched controls. Points represent individual samples; boxes indicate median and interquartile range.

**Supplementary Figure 3. Sex-dependent cytokine profiles in the placenta following maternal immune activation.** *Tnf* (A–C)*, Il1b* (D–F)*, Il6* (G-I) *and Ifng* (J-L) expression in placental tissue at 4 h , 24 h, and 48 h following maternal immune activation. Data are presented as boxplots of normalized gene expression, stratified by sex (M, male; F, female) and treatment group (C, control; LPS; Poly I:C 5 mg/kg; Poly I:C 15 mg/kg). No significant sex × treatment interaction or main effect of sex was detected at any time point. Points represent individual samples; boxes indicate median and interquartile range.

**Supplementary Figure 4. Sex-dependent cytokine profiles in the fetal brain following maternal immune activation.** *Tnf* (A–C), *Il1b* (D–F), *Il6* (G-I) and *Ifng* (J-L) expression in fetal brain tissue at 4 h, 24 h, and 48 h following maternal immune activation. Data are presented as boxplots of normalized gene expression, stratified by sex (M, male; F, female) and treatment group (C, control; LPS; Poly I:C 5 mg/kg; Poly I:C 15 mg/kg). Statistical significance is indicated by asterisks (**p* < 0.05, ***p* < 0.01) relative to sex-matched controls. Points represent individual samples; boxes indicate median and interquartile range.

**Supplementary Figure 5. Post-implantation loss following maternal immune activation.** Post-implantation loss, defined as the proportion of resorptions and dead fetuses relative to total implantation sites per dam, is shown for control (C), LPS, Poly I:C 5 mg/kg, and Poly I:C 15 mg/kg groups at 4, 24, and 48 hours following the final injection. Group differences were assessed at each timepoint using a rank-based permutation Kruskal-Wallis test, followed by Mann-Whitney permutation post hoc tests with FDR correction where the omnibus test was significant. At 48 h, post-implantation loss was significantly elevated in the Poly I:C 5 mg/kg group relative to control. *q* < 0.05 vs. control (FDR-corrected).

**Supplementary Figure 6. Decidual kynurenine-pathway gene and protein expression and multivariate structure following maternal immune activation.** (A–C) Heat maps showing normalized gene expression (z-scores) of decidual transcripts significantly altered relative to control at 4, 24, and 48 h. Transcripts include kynurenine-pathway enzymes (*Ido2*, *Kmo*, *Kynu*, *Haao*, *Qprt*, *Kat1*) and System L amino acid transporters (*Slc7a5*/Lat1, *Slc7a8*/Lat2, *Slc3a2*). (D–F) Principal component analysis (PCA) biplots of the same transcript sets at 4, 24, and 48 h. Points represent pooled decidual samples stratified by sex (F, female; M, male), and ellipses denote 95% confidence intervals for each treatment group. Arrows indicate variable loadings, reflecting the direction and relative contribution of each transcript to the principal components; axis labels indicate the percentage of variance explained by PC1 and PC2. Female and male samples cluster together within treatment groups at all time points, consistent with the absence of sex-dependent segregation or treatment × sex interaction. All variables were centered and scaled prior to PCA. (G–I) Quantification of IDO1, KAT, and KMO protein expression in decidual tissue at 4 h (G), 24 h (H), and 48 h (I) following maternal immune activation, as assessed by Western blot analysis. No consistent changes in protein expression were observed across treatment groups or time points, in agreement with the limited transcriptional response detected in this compartment. Representative blot images are provided in Additional file 2.

**Supplementary Figure 7. Placenta kynurenine-pathway protein expression following maternal immune activation.** Quantification of IDO1, KAT, and KMO protein expression in placenta tissue at 4 h, 24 h, and 48 h following maternal immune activation, as assessed by Western blot analysis. Statistical significance is indicated by asterisks (**p* < 0.05) relative to controls. Points represent individual samples; boxes indicate median and interquartile range. Representative blot images are provided in Additional file 2.

**Supplementary Table 1. List of predesigned probe assay (FAM) used in qPCR analysis.**

| **Gene name** | **Gene symbol** | **Assay ID** |
| --- | --- | --- |
| **Reference genes** |  |  |
| Glyceraldehyde-3-phosphate dehydrogenase | *Gapdh* | Rn01775763_g1 |
| TATA-box binding protein | *Tbp* | Rn01455646_m1 |
| β2 microglobulin | *B2m* | Rn00560865_m1 |
| Tyrosine3-monooxygenase/tryptophan5-monooxygenase activation protein zeta | *Ywhaz* | Rn00755072_m1 |
| **Target genes** | | |
| Interleukin 1 beta | *Il1b* | Rn00580432_m1 |
| Interleukin 6 | *Il6* | Rn01410330_m1 |
| Tumor necrosis factor | *Tnf* | Rn99999017_m1 |
| Interferon gamma | *Ifng* | Rn00594078_m1 |
| Kynureninase | *Kynu* | Rn01449532_m1 |
| 3-hydroxyanthranilate 3,4-dioxygenase | *Haao* | Rn01469327_m1 |
| Kynurenine aminotransferase 1 (KAT 1) | *Kat1* | Rn01439192_m1 |
| Kynurenine 3-monooxygenase | *Kmo* | Rn01411937_m1 |
| Quinolinate phosphoribosyltransferase | *Qprt* | Rn01506918_g1 |
| Indoleamine 2,3-dioxygenase 2 | *Ido2* | Rn01482543_m1 |
| Solute carrier family 7 member 5 (LAT 1) | *Slc7a5* | Rn00569313_m1 |
| Solute carrier family 3 member 2 (4F2HC) | *Slc3a2* | Rn01759899_g1 |
| Solute carrier family 7 member 8 (LAT 2) | *Slc7a8* | Rn00584909_m1 |

**Supplementary Table 2. Amniotic fluid cytokine concentrations following maternal immune activation.**

| **Analyte** | **4 hours** | | | | **24 hours** | | | | **48 hours** | | | | |
| --- | --- | --- | --- | --- | --- | --- | --- | --- | --- | --- | --- | --- | --- |
|  | **CTRL** | **Poly I:C 5 mg/kg** | **Poly I:C 15 mg/kg** | **LPS 1 mg/kg** | **CTRL** | **Poly I:C 5 mg/kg** | **Poly I:C 15 mg/kg** | **LPS 1 mg/kg** | **CTRL** | **Poly I:C 5 mg/kg** | **Poly I:C 15 mg/kg** | **LPS 1 mg/kg** |  |
| *IL-6* [pg/ml] | 156.0 (149.0-179.5) | 206.8 (187.2-223.3) | 207.8 (206.2-216.3) | 172.8 (151.1-211.5) | 153.5 (144.6-168.6) | 170.3 (149.4-243.7) | 174.6 (158.3-190.0) | 161.7 (147.2-178.3) | 150.2 (140.1-166.1) | 179.5 (160.5-204.2) | 179.8 (154.2-211.7) | 155.6 (149.5-164.2) |  |
| *IL-1β* [pg/ml] | 17.7 (13.0-20.6) | 50.9 (47.6-57.0) | 20.3 (19.4-60.8) | 24.5 (15.1-49.5) | 21.1 (19.7-24.2) | 47.9 (41.3-78.9) | 19.0 (15.9-43.1) | 79.1 (72.1-81.1) | 28.8 (13.2-39.9) | 47.6 (40.5-68.3) | 47.3 (32.2-51.5) | 60.9 (48.3-87.5) |  |
| *TNF-α* [pg/ml] | 3.7 (2.7-4.7) | 7.0 (6.5-8.0) * | 7.4 (6.3-8.1) | 6.0 (4.7-7.3) | 4.1 (2.7-4.7) | 7.0 (5.9-8.5) * | 3.2 (2.9-4.1) | 7.3 (6.3-7.7) * | 5.1 (1.5-6.2) | 7.2 (3.8-7.8) | 6.8 (5.6-7.7) | 4.8 (2.2-6.3) |  |
| *CRP* [ng/ml] | 112.5 (94.1-128.1) | 172.5 (159.9-192.3) * | 154.0 (133.8-165.5) | 136.2 (135.0-140.5) | 115.1 (91.0-136.9) | 123.9 (117.8-126.4) | 130.9 (111.7-142.0) | 167.8 (152.5-174.6) | 99.1 (79.5-115.8) | 162.1 (155.0-174.6) * | 158.4 (151.3-162.9) * | 146.9 (122.8-174.5) |  |

**Amniotic fluid cytokine concentrations at 4, 24, and 48 hours after** **maternal immune activation.** Data are presented as median with interquartile range (25th–75th percentile), calculated using one averaged value per dam (mean of female and male pups). Statistical comparisons were performed as described in the Methods. Significant differences relative to the control group are indicated by *p < 0.05.

**Supplementary Table 3. Cytokine gene expression in the placenta and fetal brain following maternal immune activation.**

| **Tissue** | **Gene** | **4 hours** | | | | **24 hours** | | | | **48 hours** | | | |
| --- | --- | --- | --- | --- | --- | --- | --- | --- | --- | --- | --- | --- | --- |
|  |  | **CTRL** | **LPS 1 mg/kg** | **Poly I:C 5 mg/kg** | **Poly I:C 15 mg/kg** | **CTRL** | **LPS 1 mg/kg** | **Poly I:C 5 mg/kg** | **Poly I:C 15 mg/kg** | **CTRL** | **LPS 1 mg/kg** | **Poly I:C 5 mg/kg** | **Poly I:C 15 mg/kg** |
| **Placenta** | *Il1b* | 0.38 (-0.11 to 0.65) | 1.36 (1.09 to 1.63) * | -0.10 (-0.64 to 0.11) | -0.42 (-0.90 to -0.10) * | -0.56 (-0.84 to 0.12) | 0.76 (0.45 to 1.06) * | -0.74 (-0.92 to 0.01) | 0.17 (-0.11 to 0.46) | -0.32 (-0.67 to -0.14) | 0.32 (0.08 to 0.76) | 0.72 (0.19 to 2.04) | 0.68 (0.54 to 0.98) |
|  | *Il6* | 1.65 (1.28 to 3.15) | 1.97 (1.39 to 2.29) | 2.99 (1.94 to 4.42) | 1.66 (1.50 to 1.90) | 1.92 (0.58 to 2.20) | 2.54 (2.53 to 2.80) | 2.32 (1.44 to 2.80) | 1.11 (1.05 to 1.29) | 0.53 (0.39 to 0.78) | 1.17 (-0.43 to 2.06) | 2.34 (1.89 to 2.70) | 0.20 (-0.01 to 0.97) |
|  | *Tnf* | 1.50 (0.03 to 1.75) | 2.48 (1.47 to 3.02) * | 1.16 (-0.10 to 1.81) | 2.18 (1.27 to 2.24) * | -0.66 (-1.20 to 0.34) | -0.69 (-0.82 to -0.18) | -0.42 (-0.96 to -0.26) | 1.69 (1.12 to 1.95) | -0.57 (-1.25 to -0.14) | 0.94 (-0.24 to 1.42) | -0.00 (-0.71 to 1.01) | 1.45 (1.36 to 1.90) |
|  | *Ifng* | 0.52 (-0.36 to 1.36) | 2.00 (1.70 to 2.22) * | -0.18 (-0.91 to 0.46) | 1.41 (0.84 to 1.45) | -0.74 (-1.36 to 1.00) | -0.08 (-0.63 to 0.71) | -1.69 (-2.16 to -1.23) | 1.98 (1.43 to 2.29) | -0.74 (-0.90 to -0.25) | 0.47 (-0.42 to 0.80) | -1.57 (-1.82 to -1.45) | 0.70 (0.49 to 1.15) |
| **Fetal brain** | *Il1b* | -3.24 (-3.24 to -2.96) | -3.51 (-3.79 to -3.13) | -3.47 (-4.07 to -3.35) | -3.87 (-4.14 to -3.26) | -3.08 (-3.60 to -2.87) | -3.29 (-3.52 to -3.18) | -2.91 (-3.25 to -2.49) | -3.51 (-3.62 to -3.30) | -2.96 (-3.18 to -2.61) | -4.07 (-4.25 to -3.40) | -3.06 (-3.16 to -2.53) | -3.53 (-3.64 to -3.24) |
|  | *Il6* | -1.32 (-1.92 to -0.76) | -1.46 (-1.65 to -1.12) | -0.91 (-1.07 to -0.64) | -2.14 (-2.56 to -2.02) | -0.91 (-1.34 to -0.56) | -1.43 (-1.60 to -0.78) | -0.64 (-1.02 to -0.57) | -1.90 (-2.21 to -1.31) | -0.80 (-1.20 to -0.58) | -1.76 (-2.35 to -1.32) | -0.63 (-1.01 to -0.49) | -1.02 (-1.99 to -0.92) |
|  | *Tnf* | 0.11 (-0.40 to 0.16) | -0.01 (-0.06 to -0.00) | -0.18 (-0.31 to 0.11) | -0.31 (-0.39 to -0.00) | -0.17 (-0.56 to 0.04) | -0.22 (-0.35 to -0.01) | -0.39 (-0.82 to -0.28) | -0.11 (-0.68 to 0.45) | 0.11 (0.05 to 0.26) | -1.22 (-1.53 to -0.66) * | -0.20 (-0.40 to -0.10) | -0.14 (-0.70 to 0.04) |
|  | *Ifng* | 0.29 (0.16 to 0.58) | 0.15 (-0.32 to 0.49) | -0.30 (-0.50 to -0.04) | -0.53 (-0.74 to 0.46) | -0.20 (-0.31 to 0.40) | -0.49 (-0.72 to -0.01) | 0.63 (0.36 to 1.07) | -0.36 (-0.53 to 0.45) | 0.60 (0.02 to 0.89) | -0.81 (-1.17 to -0.67) | 0.46 (0.21 to 0.66) | -0.10 (-0.78 to -0.02) |

**Placental and fetal brain cytokine transcript levels at 4, 24, and 48 hours maternal imnune activation.** Data are presented as median with interquartile range (25th–75th percentile) of log2-transformed normalized expression (NE) values. For each dam, individual female and male pup NE values were log2-transformed and averaged to yield one value per litter, which was used for descriptive statistics and statistical analysis. Statistical comparisons were performed as described in the Methods. Significant differences relative to the control group are indicated by *p < 0.05.

**Supplementary Table 4. Litter composition and pregnancy outcome by treatment group.**

| **Group** | **n dams** | **Live litter size (mean ± SEM)** | **Resorptions (n, %)** | **Dead fetuses (n, %)** | **Post-implantation loss (%)** |
| --- | --- | --- | --- | --- | --- |
| Control | 21 | 13.9 ± 0.8 | 8 (2.7) | 1 (0.3) | 3.0 |
| LPS 1 mg/kg | 20 | 12.9 ± 1.0 | 7 (2.6) | 7 (2.6) | 5.1 |
| Poly I:C 5 mg/kg | 18 | 11.3 ± 0.8 | 17 (6.3) | 47 (17.5) | 23.9 |
| Poly I:C 15 mg/kg | 19 | 12.8 ± 0.6 | 13 (4.9) | 9 (3.4) | 8.3 |

**Live litter size, resorptions, dead fetuses, and post-implantation loss per dam for each treatment group, pooled across the 4, 24, and 48 h cohorts.** Live litter size and post-implantation loss were compared across treatment groups separately at each timepoint (permutation-based tests). No significant differences were observed for litter size at any timepoint; for post-implantation loss, see Supplementary Figure 5 for statistics and timepoint-resolved distributions. The pooled values above are shown for overview.

**Supplementary Table 5. Kynurenine-pathway and System L transporter gene expression in the decidua following maternal immune activation.**

| **Gene** | **4 hours** | | | | **24 hours** | | | | **48 hours** | | | | |
| --- | --- | --- | --- | --- | --- | --- | --- | --- | --- | --- | --- | --- | --- |
|  | **CTRL** | **LPS 1 mg/kg** | **Poly I:C 5 mg/kg** | **Poly I:C 15 mg/kg** | **CTRL** | **LPS 1 mg/kg** | **Poly I:C 5 mg/kg** | **Poly I:C 15 mg/kg** | **CTRL** | **LPS 1 mg/kg** | **Poly I:C 5 mg/kg** | **Poly I:C 15 mg/kg** |  |
| *Ido2* | -0.19 (-0.39 to -0.02) | -0.06 (-0.33 to 0.13) | 0.38 (-0.09 to 0.75) | 0.26 (0.15 to 0.53) | -0.29 (-0.55 to 0.27) | -0.60 (-0.81 to -0.51) | 0.21 (-0.01 to 0.30) | -0.27 (-0.44 to -0.09) | 0.17 (0.08 to 0.28) | -0.20 (-0.52 to 0.00) | 0.03 (-0.13 to 0.15) | -0.27 (-0.35 to -0.11) |  |
| *Kat1* | -0.77 (-1.33 to -0.51) | -0.52 (-0.82 to -0.28) | -0.98 (-1.12 to -0.83) | -0.99 (-1.11 to -0.57) | -0.54 (-0.81 to -0.35) | -0.93 (-1.15 to -0.73) | -0.56 (-0.61 to -0.30) | -0.38 (-0.68 to -0.03) | -0.03 (-0.27 to 0.62) | -0.45 (-1.33 to -0.25) | 0.38 (-0.37 to 1.04) | -0.70 (-0.72 to -0.46) |  |
| *Kmo* | -2.01 (-2.18 to -1.16) | -1.11 (-1.30 to -0.93) | -1.63 (-2.28 to -1.19) | -1.28 (-1.87 to -0.16) | -1.13 (-1.36 to -0.58) | -0.84 (-1.22 to -0.19) | -0.93 (-1.28 to -0.21) | -1.55 (-1.82 to -0.88) | -0.39 (-0.76 to -0.18) | -0.54 (-0.68 to -0.36) | -0.01 (-0.47 to 0.87) | -1.67 (-2.05 to -1.59) * |  |
| *Kynu* | 1.03 (-0.31 to 1.64) | 0.54 (0.12 to 1.20) | 0.96 (0.01 to 1.05) | -0.11 (-0.59 to 0.51) | -0.58 (-0.84 to 0.09) | -0.28 (-0.74 to 1.06) | -0.00 (-0.15 to 0.39) | 0.20 (-0.17 to 1.43) | 0.98 (0.03 to 3.17) | 1.01 (0.18 to 1.47) | 0.55 (0.20 to 0.68) | 0.45 (0.06 to 0.72) |  |
| *Haao* | -0.86 (-1.15 to -0.19) | -0.27 (-0.44 to 0.50) | -0.76 (-1.53 to -0.02) | -0.83 (-1.19 to -0.68) | -0.03 (-1.33 to 0.49) | -1.87 (-2.13 to -1.44) | -0.29 (-0.70 to 0.37) | 0.06 (-0.48 to 1.07) | -0.66 (-1.08 to 0.29) | -0.80 (-1.69 to -0.34) | -0.73 (-1.28 to 0.04) | -1.33 (-1.70 to 0.53) |  |
| *Qprt* | -1.03 (-1.87 to -0.70) | -0.30 (-0.56 to -0.02) | -1.41 (-2.72 to -0.85) | -0.88 (-1.10 to -0.68) | -2.02 (-2.72 to -0.69) | -3.15 (-3.29 to -2.70) | -1.33 (-1.47 to -1.15) | 0.23 (0.13 to 0.29) * | -0.66 (-1.31 to -0.40) | -0.00 (-1.56 to 0.51) | -1.73 (-1.97 to -1.28) | 0.28 (0.10 to 0.42) |  |
| *Slc7a5* | -1.35 (-1.46 to -0.92) | -1.08 (-1.35 to -0.89) | -1.10 (-1.27 to -0.54) | -1.25 (-1.48 to -0.90) | -1.02 (-1.28 to -0.30) | -1.03 (-1.24 to -0.73) | 0.18 (-0.17 to 0.60) | -0.78 (-1.01 to -0.71) | -0.06 (-0.23 to 0.25) | -0.42 (-0.67 to -0.20) * | 0.16 (-0.21 to 0.29) | -0.73 (-0.77 to -0.65) * |  |
| *Slc7a8* | -1.42 (-2.82 to -1.11) | -0.93 (-1.42 to -0.58) | -1.84 (-3.51 to -1.37) | -1.03 (-1.30 to -0.79) | -2.76 (-3.93 to -1.37) | -3.73 (-3.90 to -2.05) | -1.50 (-2.09 to -1.23) | -0.98 (-1.19 to -0.80) * | -1.25 (-1.79 to -0.80) | -1.33 (-2.87 to -0.42) | -1.30 (-1.58 to -1.08) | -0.96 (-1.08 to -0.86) |  |
| *Slc3a2* | -1.00 (-1.23 to -0.92) | -0.73 (-1.04 to -0.54) | -0.93 (-1.10 to -0.89) | -1.13 (-1.51 to -0.77) | -0.72 (-1.38 to -0.28) | -1.33 (-1.56 to -1.25) | -0.19 (-0.38 to 0.32) | -0.65 (-0.90 to -0.41) | -0.22 (-0.40 to -0.06) | -0.63 (-1.10 to -0.35) | 0.05 (-0.04 to 0.25) | -0.89 (-1.09 to -0.65) |  |

**Decidual transcript levels at 4, 24, and 48 hours after maternal immune activation.** Data are presented as median with interquartile range (25th–75th percentile) of log2-transformed normalized expression (NE) values. For each dam, individual female and male pup NE values were log2-transformed and averaged to yield one value per litter, which was used for descriptive statistics and statistical analysis. Statistical comparisons were performed as described in the Methods. Significant differences relative to the control group are indicated by *p < 0.05.

**Supplementary Table 6. Kynurenine-pathway and System L transporter gene expression in the placenta following maternal immune activation.**

| **Gene** | **4 hours** | | | | **24 hours** | | | | **48 hours** | | | |
| --- | --- | --- | --- | --- | --- | --- | --- | --- | --- | --- | --- | --- |
|  | **CTRL** | **LPS 1 mg/kg** | **Poly I:C 5 mg/kg** | **Poly I:C 15 mg/kg** | **CTRL** | **LPS 1 mg/kg** | **Poly I:C 5 mg/kg** | **Poly I:C 15 mg/kg** | **CTRL** | **LPS 1 mg/kg** | **Poly I:C 5 mg/kg** | **Poly I:C 15 mg/kg** |
| *Ido2* | -0.22 (-0.63 to 0.08) | -0.42 (-0.73 to -0.14) | -0.28 (-1.13 to 0.59) | -0.68 (-0.87 to -0.32) | 0.72 (0.20 to 0.95) | 0.34 (0.08 to 0.87) | 0.53 (0.20 to 0.70) | -0.73 (-1.16 to -0.69) * | -0.06 (-0.19 to 0.49) | -0.42 (-0.59 to 0.38) | 0.59 (0.47 to 0.86) * | -1.26 (-1.57 to -1.15) * |
| *Kat1* | -1.01 (-1.76 to -0.45) | -0.88 (-1.18 to -0.40) | -1.87 (-2.05 to -1.65) | -1.42 (-2.06 to -1.32) | -1.73 (-1.82 to -1.39) | -0.79 (-1.96 to -0.46) | -1.77 (-1.93 to -1.21) | -1.17 (-1.59 to -0.69) | -0.88 (-1.33 to -0.52) | -1.28 (-1.52 to -0.77) | -2.28 (-2.74 to -1.86) | -1.85 (-2.52 to -1.61) |
| *Kmo* | 0.67 (0.37 to 1.18) | 1.62 (1.33 to 1.71) | 1.16 (0.87 to 1.40) | 0.64 (0.50 to 0.90) | 1.06 (0.68 to 1.17) | 2.17 (1.61 to 2.36) * | 1.82 (1.47 to 2.02) * | 0.84 (0.75 to 0.94) | 0.47 (0.14 to 0.83) | 0.72 (0.40 to 1.28) | 1.37 (1.03 to 1.64) | 0.12 (0.03 to 0.55) |
| *Kynu* | 1.18 (0.61 to 1.88) | 2.08 (1.92 to 2.36) * | 0.84 (0.51 to 1.06) | 0.69 (-0.09 to 1.32) | 0.84 (0.54 to 1.08) | 1.28 (0.66 to 1.46) | 0.84 (0.65 to 1.64) | 0.05 (-0.16 to 0.24) | 0.53 (0.33 to 1.06) | 0.62 (0.01 to 0.97) | 0.64 (0.59 to 1.47) | -0.28 (-0.46 to 0.17) |
| *Haao* | 0.70 (-0.17 to 1.05) | 1.86 (1.56 to 2.38) | 0.45 (-0.58 to 0.95) | 0.77 (0.22 to 1.38) | 0.47 (-0.47 to 1.33) | 1.04 (-0.09 to 1.44) | 0.97 (0.26 to 1.85) | 0.42 (0.06 to 0.65) | 0.12 (-0.38 to 0.33) | 0.81 (0.19 to 1.13) | -0.04 (-1.71 to 0.49) | -0.84 (-1.32 to -0.33) |
| *Qprt* | 0.24 (0.12 to 0.29) | 0.94 (0.57 to 1.48) * | -0.15 (-0.60 to 0.24) | -0.10 (-1.01 to 0.42) | 0.17 (-0.06 to 0.45) | 0.02 (-0.51 to 0.26) | 0.18 (0.04 to 0.78) | -0.31 (-0.85 to -0.11) | -0.10 (-0.50 to 0.43) | 0.60 (0.45 to 0.76) | -0.63 (-0.85 to 0.10) | -1.50 (-1.69 to -1.00) * |
| *Slc7a5* | -0.88 (-1.08 to -0.77) | -0.77 (-0.95 to -0.69) | -1.72 (-2.04 to -1.48) * | -1.53 (-1.87 to -1.22) * | -0.42 (-0.64 to -0.14) | -1.17 (-1.26 to -0.41) | -0.80 (-1.04 to -0.43) | -1.05 (-1.38 to -0.75) | 0.24 (0.03 to 0.74) | -0.03 (-0.37 to 0.08) | -0.34 (-0.76 to -0.10) * | -1.29 (-1.35 to -0.64) * |
| *Slc7a8* | -0.14 (-1.27 to 0.31) | -0.93 (-1.16 to -0.45) | -1.43 (-1.89 to -0.96) | -1.31 (-1.86 to -1.13) | -0.98 (-1.05 to 0.06) | -1.40 (-1.67 to -1.02) | -0.94 (-1.10 to -0.75) | -1.10 (-1.68 to -0.65) | 0.72 (0.50 to 0.81) | 0.99 (-0.28 to 1.25) | -0.31 (-1.02 to 0.02) * | -0.07 (-0.43 to 0.15) * |
| *Slc3a2* | -2.12 (-2.19 to -2.01) | -1.58 (-1.76 to -1.53) | -2.30 (-2.80 to -2.10) | -2.16 (-2.35 to -2.12) | -1.19 (-1.99 to -1.06) | -1.75 (-2.34 to -1.70) | -1.45 (-1.52 to -1.24) | -1.61 (-2.05 to -1.33) | -0.19 (-0.56 to -0.13) | -1.13 (-1.15 to -0.98) * | -1.52 (-1.73 to -1.03) * | -1.46 (-1.75 to -1.09) * |

**Placental transcript levels at 4, 24, and 48 hours maternal immune activation.** Data are presented as median with interquartile range (25th–75th percentile) of log2-transformed normalized expression (NE) values. For each dam, individual female and male pup NE values were log2-transformed and averaged to yield one value per litter, which was used for descriptive statistics and statistical analysis. Statistical comparisons were performed as described in the Methods. Significant differences relative to the control group are indicated by *p < 0.05.

**Supplementary Table 7. Kynurenine-pathway and System L transporter gene expression in the fetal brain following maternal immune activation.**

| **Gene** | **4 hours** | | | | **24 hours** | | | | **48 hours** | | | |
| --- | --- | --- | --- | --- | --- | --- | --- | --- | --- | --- | --- | --- |
|  | **CTRL** | **LPS 1 mg/kg** | **Poly I:C 5 mg/kg** | **Poly I:C 15 mg/kg** | **CTRL** | **LPS 1 mg/kg** | **Poly I:C 5 mg/kg** | **Poly I:C 15 mg/kg** | **CTRL** | **LPS 1 mg/kg** | **Poly I:C 5 mg/kg** | **Poly I:C 15 mg/kg** |
| *Ido2* | -0.33 (-0.68 to 0.08) | -1.07 (-1.62 to -0.82) | -0.54 (-0.63 to -0.36) | -1.28 (-1.68 to -0.87) | -0.11 (-0.46 to 0.02) | -0.52 (-0.79 to -0.32) | -0.50 (-0.56 to -0.43) | -0.88 (-1.23 to -0.37) * | -0.45 (-0.77 to -0.37) | -0.70 (-1.23 to -0.37) | -0.44 (-0.70 to -0.30) | -0.69 (-1.09 to 0.05) |
| *Kat1* | 0.37 (-0.99 to 0.67) | -0.26 (-0.31 to 0.19) | 0.40 (0.28 to 0.84) | -0.81 (-1.31 to -0.34) | 0.54 (0.50 to 1.02) | 0.05 (-0.00 to 0.56) | 0.89 (0.63 to 0.93) | -0.71 (-0.93 to -0.15) * | 0.40 (0.30 to 0.67) | -0.49 (-1.43 to 0.06) * | 0.69 (0.30 to 0.73) | -0.03 (-0.98 to 0.09) |
| *Kmo* | -0.23 (-0.96 to 1.13) | 0.12 (-0.11 to 0.82) | 1.06 (0.74 to 1.56) | -0.65 (-0.80 to 0.60) | 0.99 (0.82 to 1.07) | 0.85 (0.43 to 1.00) | 1.08 (0.72 to 1.44) | -0.18 (-0.73 to 0.10) * | 0.93 (0.62 to 1.63) | -1.24 (-1.45 to -0.16) * | 1.35 (1.02 to 1.66) | 0.20 (-0.44 to 1.05) |
| *Kynu* | -0.52 (-0.78 to 0.65) | -0.34 (-0.54 to 0.09) | 0.27 (0.10 to 1.39) | -1.03 (-1.26 to -0.35) | 0.46 (0.19 to 1.12) | 1.01 (0.56 to 1.25) | 0.80 (0.67 to 0.90) | -0.36 (-0.68 to 0.15) * | 0.98 (0.65 to 1.43) | 0.46 (-0.83 to 0.58) * | 1.28 (0.09 to 1.69) | 0.51 (-0.05 to 0.77) |
| *Haao* | -0.99 (-1.27 to -0.21) | -0.76 (-1.06 to -0.23) | -0.03 (-0.34 to 0.39) | -1.08 (-1.22 to -0.86) | 0.27 (-0.03 to 0.47) | -0.73 (-0.83 to -0.52) | -0.15 (-0.63 to 0.18) | -1.16 (-1.59 to -0.71) * | -0.45 (-0.65 to -0.05) | -0.56 (-1.02 to -0.42) | 0.09 (-0.38 to 0.48) | -0.17 (-1.09 to -0.01) |
| *Qprt* | 0.54 (-0.51 to 0.89) | -0.20 (-0.25 to 0.30) | 0.59 (0.54 to 0.88) | -0.45 (-0.85 to 0.11) | 0.56 (0.36 to 0.81) | 0.56 (0.36 to 0.67) | 0.49 (0.18 to 0.66) | -0.15 (-0.34 to 0.17) * | 0.68 (0.39 to 1.39) | 0.07 (-1.10 to 0.46) * | 0.50 (0.41 to 0.86) | 0.39 (0.02 to 0.72) |
| *Slc7a5* | -0.42 (-2.07 to -0.16) | -1.21 (-1.61 to -0.87) | -0.11 (-0.94 to 0.25) | -1.61 (-2.16 to -1.29) | -0.25 (-0.47 to 0.01) | -1.14 (-1.35 to -0.60) | -0.04 (-0.27 to 0.36) | -1.56 (-1.83 to -1.40) * | 0.12 (-0.25 to 0.18) | -1.60 (-2.10 to -0.75) * | -0.17 (-0.40 to 0.03) | -1.00 (-1.53 to -0.58) |
| *Slc7a8* | -0.35 (-2.02 to -0.29) | -1.42 (-1.63 to -0.99) | -0.80 (-1.10 to -0.50) | -1.75 (-1.85 to -1.40) | -0.74 (-0.88 to -0.51) | -1.21 (-1.41 to -1.06) | -0.32 (-0.64 to -0.10) | -1.69 (-1.86 to -1.45) * | -0.29 (-0.96 to -0.10) | -1.85 (-2.63 to -1.15) * | -0.29 (-0.61 to 0.15) | -0.88 (-1.50 to -0.50) |
| *Slc3a2* | -0.56 (-1.70 to -0.26) | -1.35 (-1.61 to -0.89) | -0.36 (-0.62 to -0.25) | -1.68 (-2.01 to -1.36) | -0.31 (-0.46 to -0.12) | -0.91 (-0.95 to -0.72) * | -0.18 (-0.45 to -0.07) | -1.47 (-1.76 to -1.24) * | -0.28 (-0.38 to -0.18) | -1.65 (-2.15 to -0.92) * | -0.29 (-0.50 to -0.13) | -0.95 (-1.71 to -0.53) |

**Fetal brain transcript levels at 4, 24, and 48 hours after maternal immune activation.** Data are presented as median with interquartile range (25th–75th percentile) of log2-transformed normalized expression (NE) values. For each dam, individual female and male pup NE values were log2-transformed and averaged to yield one value per litter, which was used for descriptive statistics and statistical analysis. Statistical comparisons were performed as described in the Methods. Significant differences relative to the control group are indicated by *p < 0.05.

**Supplementary Table 8. Within-litter placenta–fetal brain transcript correlations for kynurenine-pathway genes.**

A) Across all samples

| **Gene** | **Partial Spearman ρ** | **q-value** |
| --- | --- | --- |
| *Kmo* | +0.27 | 0.0045 |
| *Kat1* | −0.26 | 0.0045 |
| *Qprt* | −0.15 | 0.140 |
| *Ido2* | +0.07 | 0.528 |
| *Haao* | +0.03 | 0.864 |
| *Kynu* | +0.01 | 0.944 |

B) Within each treatment group (Kmo and Kat1 only)

| **Gene** | **Treatment** | **Partial Spearman ρ** | **q-value** |
| --- | --- | --- | --- |
| *Kmo* | Control | +0.24 | 0.261 |
| *Kmo* | LPS | +0.37 | 0.157 |
| *Kmo* | Poly I:C 5 mg/kg | +0.23 | 0.261 |
| *Kmo* | Poly I:C 15 mg/kg | −0.01 | 0.966 |
| *Kat1* | Control | −0.43 | 0.019 |
| *Kat1* | LPS | −0.04 | 0.851 |
| *Kat1* | Poly I:C 5 mg/kg | −0.10 | 0.787 |
| *Kat1* | Poly I:C 15 mg/kg | −0.52 | 0.006 |

**Partial Spearman rank correlations between placental and fetal-brain transcript levels for the six kynurenine-pathway genes quantified in both compartments, based on 150 litter-matched placenta–brain pairs from 78 litters across all three post-injection time points*.*** Correlations were adjusted for treatment group, time point, and fetal sex, and statistical significance was assessed by a restricted permutation procedure (10,000 permutations) with whole litters exchanged as blocks; p-values were FDR-corrected across the six genes (q < 0.05 considered significant). Within-treatment correlations (adjusting for time and sex) are shown only for *Kmo* and *Kat1*, the two genes showing a significant overall placenta–brain correlation; these subgroup analyses were underpowered (~17–20 litters per group) and are presented as supporting data. The placenta × treatment interaction was non-significant for all six genes.
