# Supplementary material for: Maternal immune activation disrupts the kynurenine pathway of tryptophan metabolism in the placenta–fetal brain axis in rats
Source: Mol Med. 2026 Aug 7;32:214. doi: 10.1186/s10020-026-01587-1 (PMC13749810; doi:10.1186/s10020-026-01587-1)
Supplement: Supplementary file 2 [file 10020_2026_1587_MOESM2_ESM.docx]

# Representative Western Blots Images

# This file provides representative western blot images supporting the quantification presented in Supplementary Figures 6 and 7. For each protein, tissue, sex, and time point, one representative membrane is shown, comprising the target protein (A), the loading control, Actin (B), and the molecular weight marker (C). Lane assignments for each membrane are indicated in the accompanying table (Control; PIC 5, Poly I:C 5 mg/kg; PIC 15, Poly I:C 15 mg/kg; LPS, 1 mg/kg; Calib., calibrator sample). Membranes are shown uncropped to allow assessment of band specificity

### 1. Placentas

### A. IDO 1:

### 4 h treatment:

### *Females:*

| **A) IDO**   | **B) Actin**   | **C) Molecular Weight Marker**   |
| --- | --- | --- |

| **Lane** | 1 | 2 | 3 | 4 | 5 | 6 | 7 | 8 | 9 | 10 | 11 | 12 | 13 |
| --- | --- | --- | --- | --- | --- | --- | --- | --- | --- | --- | --- | --- | --- |
| **Group** | Control | Control | Control | PIC 5 | PIC 5 | PIC 5 | PIC 15 | PIC 15 | PIC 15 | LPS | LPS | LPS | Calib. |

### *Males:*

| **A) IDO**   | **B) Actin**   | **C) Molecular Weight Marker**   |
| --- | --- | --- |

| **Lane** | 1 | 2 | 3 | 4 | 5 | 6 | 7 | 8 | 9 | 10 | 11 | 12 | 13 |
| --- | --- | --- | --- | --- | --- | --- | --- | --- | --- | --- | --- | --- | --- |
| **Group** | Control | Control | Control | PIC 5 | PIC 5 | PIC 5 | PIC 15 | PIC 15 | PIC 15 | LPS | LPS | LPS | Calib. |

### 24 h treatment:

### *Females:*

| **A) IDO**   | **B) Actin**   | **C) Molecular Weight Marker**   |
| --- | --- | --- |

| **Lane** | 1 | 2 | 3 | 4 | 5 | 6 | 7 | 8 | 9 | 10 | 11 | 12 | 13 |
| --- | --- | --- | --- | --- | --- | --- | --- | --- | --- | --- | --- | --- | --- |
| **Group** | Control | Control | Control | Control | Calib. | PIC 5 | PIC 5 | PIC 15 | PIC 15 | PIC 15 | LPS | LPS | LPS |

### *Males:*

| **A) IDO**   | **B) Actin**   | **C) Molecular Weight Marker**   |
| --- | --- | --- |

| **Lane** | 1 | 2 | 3 | 4 | 5 | 6 | 7 | 8 | 9 | 10 | 11 | 12 | 13 |
| --- | --- | --- | --- | --- | --- | --- | --- | --- | --- | --- | --- | --- | --- |
| **Group** | Control | Control | Control | Calib. | PIC 5 | PIC 5 | PIC 5 | PIC 15 | PIC 15 | PIC 15 | LPS | LPS | LPS |

### 48 h treatment:

### *Females:*

| **A) IDO**   | **B) Actin**   | **C) Molecular Weight Marker**   |
| --- | --- | --- |

| **Lane** | 1 | 2 | 3 | 4 | 5 | 6 | 7 | 8 | 9 | 10 | 11 | 12 | 13 |
| --- | --- | --- | --- | --- | --- | --- | --- | --- | --- | --- | --- | --- | --- |
| **Group** | Control | Control | Control | Calib. | PIC 5 | PIC 5 | PIC 5 | PIC 15 | PIC 15 | PIC 15 | LPS | LPS | LPS |

### *Males:*

| **A) IDO**   | **B) Actin**   | **C) Molecular Weight Marker**   |
| --- | --- | --- |

| **Lane** | 1 | 2 | 3 | 4 | 5 | 6 | 7 | 8 | 9 | 10 | 11 | 12 | 13 | 14 |
| --- | --- | --- | --- | --- | --- | --- | --- | --- | --- | --- | --- | --- | --- | --- |
| **Group** | Control | Control | Control | Calib. | PIC 5 | PIC 5 | PIC 5 | PIC 15 | PIC 15 | PIC 15 | PIC 15 | LPS | LPS | LPS |

### B. KMO:

### 4 h treatment:

### *Females:*

| **A) KMO**   | **B) Actin**   | **C) Molecular Weight Marker**   |
| --- | --- | --- |

| **Lane** | 1 | 2 | 3 | 4 | 5 | 6 | 7 | 8 | 9 | 10 | 11 | 12 | 13 |
| --- | --- | --- | --- | --- | --- | --- | --- | --- | --- | --- | --- | --- | --- |
| **Group** | Control | Control | Control | Calib. | PIC 5 | PIC 5 | PIC 5 | PIC 15 | PIC 15 | PIC 15 | LPS | LPS | LPS |

### *Males:*

| **A) KMO**   | **B) Actin**   | **C) Molecular Weight Marker**   |
| --- | --- | --- |

| **Lane** | 1 | 2 | 3 | 4 | 5 | 6 | 7 | 8 | 9 | 10 | 11 | 12 | 13 |
| --- | --- | --- | --- | --- | --- | --- | --- | --- | --- | --- | --- | --- | --- |
| **Group** | Control | Control | Control | PIC 5 | PIC 5 | PIC 5 | PIC 15 | PIC 15 | PIC 15 | LPS | Calib. | LPS | LPS |

### 24 h treatment:

### *Females:*

| **A) KMO**   | **B) Actin**   | **C) Molecular Weight Marker**   |
| --- | --- | --- |

| **Lane** | 1 | 2 | 3 | 4 | 5 | 6 | 7 | 8 | 9 | 10 | 11 | 12 | 13 |
| --- | --- | --- | --- | --- | --- | --- | --- | --- | --- | --- | --- | --- | --- |
| **Group** | Control | Control | Control | Calib. | PIC 5 | PIC 5 | PIC 5 | PIC 15 | PIC 15 | PIC 15 | LPS | LPS | LPS |

### *Males:*

| **A) KMO**   | **B) Actin**   | **C) Molecular Weight Marker**   |
| --- | --- | --- |

| **Lane** | 1 | 2 | 3 | 4 | 5 | 6 | 7 | 8 | 9 | 10 | 11 | 12 | 13 |
| --- | --- | --- | --- | --- | --- | --- | --- | --- | --- | --- | --- | --- | --- |
| **Group** | Control | Control | Control | Calib. | PIC 5 | PIC 5 | PIC 5 | PIC 15 | PIC 15 | PIC 15 | LPS | LPS | LPS |

### 48 h treatment:

### *Females:*

| **A) KMO**   | **B) Actin**   | **C) Molecular Weight Marker**   |
| --- | --- | --- |

| **Lane** | 1 | 2 | 3 | 4 | 5 | 6 | 7 | 8 | 9 | 10 | 11 | 12 | 13 |
| --- | --- | --- | --- | --- | --- | --- | --- | --- | --- | --- | --- | --- | --- |
| **Group** | Control | Control | Control | Calib. | PIC 5 | PIC 5 | PIC 5 | PIC 15 | PIC 15 | PIC 15 | LPS | LPS | LPS |

### *Males:*

| **A) KMO**   | **B) Actin**   | **C) Molecular Weight Marker**   |
| --- | --- | --- |

| **Lane** | 1 | 2 | 3 | 4 | 5 | 6 | 7 | 8 | 9 | 10 | 11 | 12 | 13 |
| --- | --- | --- | --- | --- | --- | --- | --- | --- | --- | --- | --- | --- | --- |
| **Group** | Control | Control | Control | Calib. | PIC 5 | PIC 5 | PIC 5 | PIC 15 | PIC 15 | PIC 15 | LPS | LPS | LPS |

### C. KAT:

### 4 h treatment:

### *Females:*

| **A) KAT**   | **B) Actin**   | **C) Molecular Weight Marker**   |
| --- | --- | --- |

| **Lane** | 1 | 2 | 3 | 4 | 5 | 6 | 7 | 8 | 9 | 10 | 11 | 12 | 13 |
| --- | --- | --- | --- | --- | --- | --- | --- | --- | --- | --- | --- | --- | --- |
| **Group** | Control | Control | Control | Calib. | PIC 5 | PIC 5 | PIC 5 | PIC 15 | PIC 15 | PIC 15 | LPS | LPS | LPS |

### *Males:*

| **A) KAT**   | **B) Actin**   | **C) Molecular Weight Marker**   |
| --- | --- | --- |

| **Lane** | 1 | 2 | 3 | 4 | 5 | 6 | 7 | 8 | 9 | 10 | 11 | 12 | 13 |
| --- | --- | --- | --- | --- | --- | --- | --- | --- | --- | --- | --- | --- | --- |
| **Group** | Control | Control | Control | Calib. | PIC 5 | PIC 5 | PIC 5 | PIC 15 | PIC 15 | PIC 15 | LPS | LPS | LPS |

### 24 h treatment:

### *Females*:

| **A) KAT**   | **B) Actin**   | **C) Molecular Weight Marker**   |
| --- | --- | --- |

| **Lane** | 1 | 2 | 3 | 4 | 5 | 6 | 7 | 8 | 9 | 10 | 11 | 12 | 13 |
| --- | --- | --- | --- | --- | --- | --- | --- | --- | --- | --- | --- | --- | --- |
| **Group** | Control | Control | Control | Control | Calib. | PIC 5 | PIC 5 | PIC 15 | PIC 15 | PIC 15 | LPS | LPS | LPS |

### *Males:*

| **A) KAT**   | **B) Actin**   | **C) Molecular Weight Marker**   |
| --- | --- | --- |

| **Lane** | 1 | 2 | 3 | 4 | 5 | 6 | 7 | 8 | 9 | 10 | 11 | 12 | 13 |
| --- | --- | --- | --- | --- | --- | --- | --- | --- | --- | --- | --- | --- | --- |
| **Group** | Control | Control | Control | Calib. | PIC 5 | PIC 5 | PIC 5 | PIC 15 | PIC 15 | PIC 15 | LPS | LPS | LPS |

### 48 h treatment:

### *Females:*

| **A) KAT** | **B) Actin**   | **C) Molecular Weight Marker**   |
| --- | --- | --- |

| **Lane** | 1 | 2 | 3 | 4 | 5 | 6 | 7 | 8 | 9 | 10 | 11 | 12 | 13 |
| --- | --- | --- | --- | --- | --- | --- | --- | --- | --- | --- | --- | --- | --- |
| **Group** | Control | Control | Control | Calib. | PIC 5 | PIC 5 | PIC 5 | PIC 15 | PIC 15 | PIC 15 | LPS | LPS | LPS |

### *Males:*

| **A) KAT**   | **B) Actin**   | **C) Molecular Weight Marker**   |
| --- | --- | --- |

| **Lane** | 1 | 2 | 3 | 4 | 5 | 6 | 7 | 8 | 9 | 10 | 11 | 12 | 13 |
| --- | --- | --- | --- | --- | --- | --- | --- | --- | --- | --- | --- | --- | --- |
| **Group** | Control | Control | Control | Calib. | PIC 5 | PIC 5 | PIC 5 | PIC 15 | PIC 15 | PIC 15 | LPS | LPS | LPS |

### 2. Deciduas

### A. IDO 1:

### 4 h treatment:

### *Females:*

| **A) IDO**   | **B) Actin**   | **C) Molecular Weight Marker** |
| --- | --- | --- |

| **Lane** | 1 | 2 | 3 | 4 | 5 | 6 | 7 | 8 | 9 | 10 | 11 | 12 | 13 |
| --- | --- | --- | --- | --- | --- | --- | --- | --- | --- | --- | --- | --- | --- |
| **Group** | Control | Control | Control | PIC 5 | PIC 5 | PIC 5 | PIC 15 | PIC 15 | PIC 15 | LPS | LPS | LPS | Calib. |

### *Males:*

| **A) IDO**   | **B) Actin**   | **C) Molecular Weight Marker**   |
| --- | --- | --- |

| **Lane** | 1 | 2 | 3 | 4 | 5 | 6 | 7 | 8 | 9 | 10 | 11 | 12 |
| --- | --- | --- | --- | --- | --- | --- | --- | --- | --- | --- | --- | --- |
| **Group** | Control | Control | Control | PIC 5 | PIC 5 | PIC 15 | PIC 15 | PIC 15 | LPS | LPS | LPS | Calib. |

### 24 h treatment:

### *Females:*

| **A) IDO**   | **B) Actin**   | **C) Molecular Weight Marker**   |
| --- | --- | --- |

| **Lane** | 1 | 2 | 3 | 4 | 5 | 6 | 7 | 8 | 9 | 10 | 11 | 12 | 13 |
| --- | --- | --- | --- | --- | --- | --- | --- | --- | --- | --- | --- | --- | --- |
| **Group** | LPS | LPS | LPS | PIC 15 | PIC 15 | PIC 15 | PIC 5 | PIC 5 | PIC 5 | Calib. | Control | Control | Control |

### *Males:*

| **A) IDO**   | **B) Actin**   | **C) Molecular Weight Marker**   |
| --- | --- | --- |

| **Lane** | 1 | 2 | 3 | 4 | 5 | 6 | 7 | 8 | 9 | 10 | 11 |
| --- | --- | --- | --- | --- | --- | --- | --- | --- | --- | --- | --- |
| **Group** | Control | Control | Control | Calib. | PIC 5 | PIC 5 | PIC 15 | PIC 15 | PIC 15 | LPS | LPS |

### 48 h treatment:

### *Females:*

| **A) IDO**   | **B) Actin**   | **C) Molecular Weight Marker**   |
| --- | --- | --- |

| **Lane** | 1 | 2 | 3 | 4 | 5 | 6 | 7 | 8 | 9 | 10 | 11 | 12 |
| --- | --- | --- | --- | --- | --- | --- | --- | --- | --- | --- | --- | --- |
| **Group** | Control | Control | Control | Calib. | PIC 5 | PIC 5 | PIC 15 | PIC 15 | PIC 15 | LPS | LPS | LPS |

### *Males*:

| **A) IDO**   | **B) Actin**   | **C) Molecular Weight Marker**   |
| --- | --- | --- |

| **Lane** | 1 | 2 | 3 | 4 | 5 | 6 | 7 | 8 | 9 | 10 | 11 | 12 |
| --- | --- | --- | --- | --- | --- | --- | --- | --- | --- | --- | --- | --- |
| **Group** | Control | Control | Control | Calib. | PIC 5 | PIC 5 | PIC 15 | PIC 15 | PIC 15 | LPS | LPS | LPS |

### B. KMO:

### 4 h treatment:

### *Females:*

| **A) KMO**   | **B) Actin**   | **C) Molecular Weight Marker**   |
| --- | --- | --- |

| **Lane** | 1 | 2 | 3 | 4 | 5 | 6 | 7 | 8 | 9 | 10 | 11 | 12 |
| --- | --- | --- | --- | --- | --- | --- | --- | --- | --- | --- | --- | --- |
| **Group** | Control | Control | Control | Calib. | PIC 5 | PIC 5 | PIC 15 | PIC 15 | PIC 15 | LPS | LPS | LPS |

### *Males:*

| **A) KMO**   | **B) Actin**   | **C) Molecular Weight Marker**   |
| --- | --- | --- |

| **Lane** | 1 | 2 | 3 | 4 | 5 | 6 | 7 | 8 | 9 | 10 | 11 | 12 |
| --- | --- | --- | --- | --- | --- | --- | --- | --- | --- | --- | --- | --- |
| **Group** | Control | Control | Control | Calib. | PIC 5 | PIC 5 | PIC 15 | PIC 15 | PIC 15 | LPS | LPS | LPS |

### 24 h treatment:

### *Females:*

| **A) KMO**   | **B) Actin**   | **C) Molecular Weight Marker**   |
| --- | --- | --- |

| **Lane** | 1 | 2 | 3 | 4 | 5 | 6 | 7 | 8 | 9 | 10 | 11 | 12 | 13 |
| --- | --- | --- | --- | --- | --- | --- | --- | --- | --- | --- | --- | --- | --- |
| **Group** | Control | Control | Control | Calib. | PIC 5 | PIC 5 | PIC 5 | PIC 15 | PIC 15 | PIC 15 | LPS | LPS | LPS |

### *Males:*

| **A) KMO**   | **B) Actin**   | **C) Molecular Weight Marker**   |
| --- | --- | --- |

| **Lane** | 1 | 2 | 3 | 4 | 5 | 6 | 7 | 8 | 9 | 10 | 11 |
| --- | --- | --- | --- | --- | --- | --- | --- | --- | --- | --- | --- |
| **Group** | Control | Control | Control | Calib. | PIC 5 | PIC 5 | PIC 15 | PIC 15 | PIC 15 | LPS | LPS |

### 48 h treatment:

### *Females*:

| **A) KMO**   | **B) Actin**   | **C) Molecular Weight Marker**   |
| --- | --- | --- |

| **Lane** | 1 | 2 | 3 | 4 | 5 | 6 | 7 | 8 | 9 |
| --- | --- | --- | --- | --- | --- | --- | --- | --- | --- |
| **Group** | Control | Control | Calib. | PIC 5 | PIC 15 | PIC 15 | PIC 15 | LPS | LPS |

### *Males*:

| **A) KMO**   | **B) Actin**   | **C) Molecular Weight Marker**   |
| --- | --- | --- |

| **Lane** | 1 | 2 | 3 | 4 | 5 | 6 | 7 | 8 | 9 | 10 | 11 | 12 |
| --- | --- | --- | --- | --- | --- | --- | --- | --- | --- | --- | --- | --- |
| **Group** | Control | Control | Control | Calib. | PIC 5 | PIC 5 | PIC 15 | PIC 15 | PIC 15 | LPS | LPS | LPS |

### C. KAT:

### 4 h treatment:

### *Females:*

| **A) KAT**   | **B) Actin**   | **C) Molecular Weight Marker**   |
| --- | --- | --- |

| **Lane** | 1 | 2 | 3 | 4 | 5 | 6 | 7 | 8 | 9 | 10 | 11 | 12 |
| --- | --- | --- | --- | --- | --- | --- | --- | --- | --- | --- | --- | --- |
| **Group** | Control | Control | Control | Calib. | PIC 5 | PIC 5 | PIC 15 | PIC 15 | PIC 15 | LPS | LPS | LPS |

### *Males:*

| **A) KAT**   | **B) Actin**   | **C) Molecular Weight Marker**   |
| --- | --- | --- |

| **Lane** | 1 | 2 | 3 | 4 | 5 | 6 | 7 | 8 | 9 | 10 | 11 | 12 |
| --- | --- | --- | --- | --- | --- | --- | --- | --- | --- | --- | --- | --- |
| **Group** | Control | Control | Control | Calib. | PIC 5 | PIC 5 | PIC 15 | PIC 15 | PIC 15 | LPS | LPS | LPS |

### 24 h treatment:

### *Females:*

| **A) KAT**   | **B) Actin**   | **C) Molecular Weight Marker**   |
| --- | --- | --- |

| **Lane** | 1 | 2 | 3 | 4 | 5 | 6 | 7 | 8 | 9 | 10 | 11 |
| --- | --- | --- | --- | --- | --- | --- | --- | --- | --- | --- | --- |
| **Group** | Control | Control | Control | Control | Calib. | PIC 5 | PIC 5 | PIC 15 | PIC 15 | LPS | LPS |

### *Males*:

| **A) KAT** | **B) Actin** | **C) Molecular Weight Marker** |
| --- | --- | --- |

| **Lane** | 1 | 2 | 3 | 4 | 5 | 6 | 7 | 8 | 9 | 10 | 11 | 12 | 13 | 14 |
| --- | --- | --- | --- | --- | --- | --- | --- | --- | --- | --- | --- | --- | --- | --- |
| **Group** | Control | Control | Control | Control | Calib. | PIC 5 | PIC 5 | PIC 5 | PIC 15 | PIC 15 | PIC 15 | LPS | LPS | LPS |

### 48 h treatment:

### *Females:*

| **A) KAT** | **B) Actin** | **C) Molecular Weight Marker** |
| --- | --- | --- |

| **Lane** | 1 | 2 | 3 | 4 | 5 | 6 | 7 | 8 | 9 | 10 | 11 | 12 |
| --- | --- | --- | --- | --- | --- | --- | --- | --- | --- | --- | --- | --- |
| **Group** | Control | Control | Control | Calib. | PIC 5 | PIC 5 | PIC 15 | PIC 15 | PIC 15 | LPS | LPS | LPS |

### *Males:*

| **A) KAT** | **B) Actin** | **C) Molecular Weight Marker** |
| --- | --- | --- |

| **Lane** | 1 | 2 | 3 | 4 | 5 | 6 | 7 | 8 | 9 | 10 | 11 | 12 |
| --- | --- | --- | --- | --- | --- | --- | --- | --- | --- | --- | --- | --- |
| **Group** | Control | Control | Control | Calib. | PIC 5 | PIC 5 | PIC 15 | PIC 15 | PIC 15 | LPS | LPS | LPS |

### Control: Control untreated dam

### PIC 5: Poly I:C 5 mg/kg

### PIC 15: Poly I:C 15 mg/kg

### LPS: LPS1mg/kg

### Calib: Calibrator sample
